# Tuberculosis comorbidities, multimorbidity and mortality amongst adults in South Africa: A prospective cohort study nested in a trial

**DOI:** 10.1371/journal.pgph.0006720

**Published:** 2026-08-05

**Authors:** Greta K. Wood, Rachel L. Byrne, Limakatso Lebina, Eve Worrall, Pattamukkil Abraham, Emily L. Webb, Peter MacPherson, Neil A. Martinson, Tom Wingfield

**Affiliations:** 1 Centre for Tuberculosis Research, Department of Clinical Sciences, Liverpool School of Tropical Medicine, Liverpool, United Kingdom; 2 Department of Clinical Infection, Microbiology and Immunology, Institute of Infection, Veterinary and Ecological Sciences, University of Liverpool, Liverpool, United Kingdom; 3 Department of Tropical Medicine and Infectious Diseases, University Hospitals of Liverpool Group, Liverpool, United Kingdom; 4 Perinatal HIV Research Unit (PHRU), Faculty of Health Sciences, University of the Witwatersrand, Johannesburg, South Africa; 5 Clinical Trials Unit, Africa Health Research Institute, Durban, South Africa; 6 Global Health Economics and Financing Unit, Department of Clinical Sciences, Liverpool School of Tropical Medicine, Liverpool, United Kingdom; 7 Medical Research Council International Statistics and Epidemiology Group, Department of Infectious Disease Epidemiology and International Health, London School of Hygiene and Tropical Medicine, London, United Kingdom; 8 School of Health and Wellbeing, University of Glasgow, Glasgow, United Kingdom; 9 Department of Global Public Health, Karolinska Institutet, Stockholm, Sweden; University of Sydney, AUSTRALIA

## Abstract

Tuberculosis (TB) comorbidities and multimorbidity - the presence of two or more concurrent diseases or health conditions - are major contributors to TB incidence and mortality. We investigated the prevalence and associated mortality of TB comorbidities and multimorbidity in South Africa. We conducted a prospective cohort study of adults ≥15 years with bacteriologically-confirmed pulmonary TB, nested within a trial in two provinces of South Africa. We investigated five TB key comorbidities (HIV, undernutrition, diabetes, smoking and alcohol use), and assessed all-cause mortality over 15 months using Cox regression. TB comorbidity was defined as TB with one comorbidity, and TB multimorbidity as TB with two or more comorbidities. Of 1997 participants, 1896 (95%) had mortality data available at 15 months. Median age was 38 (IQR 29–48) years, 1203/1997 (60%) were male, 86·2% (95% CI 84·6-86·6) had TB comorbidity and 47·5% (45·3-49·7) had TB multimorbidity. TB comorbidity peaked in participants aged 40–49 years (93% [90–95]), was more prevalent in men vs women (90%[88–91] vs 81%[78–84], p < 0·001), and in urban vs rural areas (88%[86–90] vs 84%[81–86], p = 0·005). Mortality was higher for participants with TB multimorbidity with two (16·0%[13·0-18·8], HR 1·88[1·20-2·95], p = 0·006) and three or more (19·9%[14·9-24·6], HR 2·58[1·56-4·24], p < 0·001) TB key comorbidities than those with no comorbidities (11·2%[7·17-15·0]) or TB comorbidity (10·6% [8·32-12·8]). The joint population attributable fraction for the TB key comorbidities on mortality was 60·0% (49·6-69·2%). TB comorbidities and multimorbidity are highly prevalent and significantly associated with mortality. People with TB should be offered screening and management of comorbidities to reduce their risk of death.

## Introduction

Multimorbidity, the presence of two or more concurrent diseases or health conditions in a single individual, is an emerging global public health challenge [1]. As populations age and healthcare advances prolong life expectancy, large numbers of people have multiple diseases or health conditions, known as comorbidities, simultaneously. Non-communicable disease multimorbidity is estimated to affect one-in-three people in low- and middle-income countries (LMICs) [2]. This phenomenon complicates treatment strategies, amplifies healthcare costs, and worsens the outcomes of those affected, particularly within resource-constrained health systems [3].

Tuberculosis (TB), the leading infectious cause of death worldwide, presents a unique challenge within the context of comorbidities and multimorbidity [4]. While TB is preventable and curable, it continues to affect millions globally, particularly in LMICs, and is associated with a substantial and persistent burden of post-TB disability [5]. The World Health Organization (WHO) defines five key comorbidities for TB: diabetes, human immunodeficiency virus (HIV), smoking, undernutrition and disorders due to alcohol use [3]. In 2020, these five WHO-defined key comorbidities were estimated to account for 4·5 million (45%) new and relapse TB episodes globally [6]. In South Africa, the leading natural cause of death from 2016-2018 was TB, recently superseded by diabetes in 2019 [7]. However, the interaction between TB, diabetes and other comorbidities including HIV is poorly described, as are sex differences.

There are complexities in applying the concept of multimorbidity to TB disease, which have resulted in divergent case definitions and a subsequent inability to meaningfully compare data from different settings. Most notably, when defining multimorbidity as “the presence of two or more concurrent diseases or health conditions in a single individual”, there is inconsistency as to whether TB is included as one of these two diseases, or whether multimorbidity in the context of TB disease is defined as TB plus two or more concurrent diseases or health conditions. To address this, the WHO proposed the following definitions; “Comorbidity: A concurrent disease or health condition in a person with tuberculosis (TB)”, and, “Multimorbidity: The presence of two or more concurrent diseases or health conditions in a person with TB” [3]. Applying these definitions to existing and future datasets enables harmonised international research and evidence synthesis.

Identifying critical factors that contribute to adverse outcomes associated with TB comorbidities and multimorbidity, provides evidence to optimize healthcare delivery through strategies including integrated, people-centred TB services and more efficient universal healthcare systems. Addressing TB comorbidities is essential to end the TB epidemic and has been recognised as a priority in the landmark WHO Framework for Collaborative Action on TB and Comorbidities [3]. This study explores the burden and mortality of TB comorbidities and multimorbidity in South Africa.

## Methods

### Ethics statement

The University of Witwatersrand Human Research Ethics Committee (Medical) (REC-250208–004, reference: 160414) and the London School of Hygiene and Tropical Medicine granted ethical approval. Informed consent was obtained from participants or their next-of-kin. Those <18 years additionally had parental consent.

### Study setting

We conducted a prospective cohort study nested within a cluster-randomised trial of household TB contact person investigations (HomeACF). Trial methods have been previously reported [8]. We analysed data from adult participants with bacteriologically-confirmed pulmonary TB (sputum smear, Xpert, culture or histology), recruited from December 2016 through March 2019 from health centres and hospitals in Limpopo and Free State Provinces South Africa (Methods A in S1 Appendix) [8]. The Mangaung Municipality in Free State Province is predominantly urban, with an annual TB incidence at the time of the study in 2019 of 476/100,000 people per year and an antenatal HIV prevalence of 32%. Capricorn Health District is more rural, with an annual TB incidence of 201/100,000 people per year and an antenatal HIV prevalence of 22% [8]. STROBE guidelines were followed in the reporting of the study.

### Index cases and inclusion criteria

Adults (≥15 years) were eligible if they had been diagnosed with bacteriologically-confirmed pulmonary TB at any health facility in study districts in the six weeks prior to recruitment. Index cases were identified through routine reporting of TB cases in health facilities, and lists of specimens positive for TB generated by routine public sector laboratories in the two study sites. Informed consent was obtained from the index case or their next-of-kin if the index case was unable to provide informed consent. Those <18 years of age had parental consent to participate in research, and also provided their own assent. Using sensitive approaches, consent for enrolment was additionally obtained from next-of-kin of eligible patients who were recently deceased shortly after a specimen was taken that confirmed TB diagnosis. Full inclusion and exclusion criteria are provided in supplementary material (Methods B in S1 Appendix). The participants in this analysis were the index cases in the trial and a sample size calculation was therefore not performed for this analysis.

### Study procedures

At recruitment, demographic, socioeconomic, and multimorbidity data were collected from participants or their representative using a standardised questionnaire. Random finger prick glucose levels were measured in all participants ≥30 years of age. Blood pressure (BP) was measured in all participants ≥25 years, using a standardised method, at least three times with the participant seated, using an electronic calibrated device with an interval of at least three minutes between each reading. Participants diagnosed with diabetes or hypertension were referred for care at their local clinic. Blood pressure and blood glucose were not collected from deceased participants. A household visit was completed 15 months after recruitment and mortality data were collected, recording TB treatment status, HIV status and antiretroviral therapy use, and date of death.

### Definitions

These study definitions are adapted from the WHO Framework for Collaborative Action on TB and Comorbidities [3].

- Health-related risk factor: A condition, disease or behaviour that increases the likelihood of developing TB.
- Comorbidity: A concurrent disease or health condition in a person with TB.
- Multimorbidity: The presence of two or more concurrent diseases or health conditions in a person with TB.
- TB Key Comorbidities: The WHO five ‘Key drivers for TB’; diabetes, disorders due to alcohol use, HIV, smoking and undernutrition. These are defined by WHO as both the five key health-related risk factors for TB and the five comorbidities associated with poorer TB treatment outcomes.

Therefore, the term “TB comorbidity” is used to describe TB with one comorbidity, and the term “TB multimorbidity” is used to describe TB with two or more comorbidities.

Case definitions for the WHO five TB key comorbidities in this study are included in Text Box 1. Alcohol use was measured in place of alcohol use disorder. Based upon measured blood pressure, hypotension was defined as systolic BP (SBP) <90 or diastolic BP (DBP) <60 mmHg. Grade 1 hypertension was defined as SBP ≥ 140 or DBP ≥ 90 mmHg, grade 2 hypertension as SBP ≥ 160 or DBP ≥ 100mmHg, and grade 3 hypertension as SBP ≥ 180 or DBP ≥ 110mmHg.

**Box 1. Five TB key comorbidities case definitions**

Diabetes

In all participants, elevated and borderline random finger-prick blood glucose were defined as >11·1 and 7·0-11·1mmol/L respectively. In those with a diabetes diagnosis, uncontrolled diabetes was defined as random finger-prick blood glucose >11·1 mmol/L [9,10].

Diabetes comorbidity included diabetes reported on the standardised questionnaire, or elevated random blood glucose >11·1mmol/L.

Alcohol use

Alcohol use comorbidity was defined as any consumption of alcohol reported on a standardised questionnaire.

HIV

Uncontrolled HIV was defined as untreated HIV (not reported to be receiving antiretroviral therapy [ART]). Advanced HIV disease was defined as treated HIV (receiving ART) with most recent CD4 count <200 cells/mm^3^. Viral load data were not available.

Smoking

Smoking was defined as being a current smoker reported on a standardised questionnaire.

Undernutrition

Undernutrition was defined as body mass index (BMI) category ‘underweight’ < 18·5 kg/m^2^. Severe undernutrition was defined as BMI category ‘severely underweight’ < 16·5 kg/m^2^. For participants enrolled after death, undernutrition was defined as premortem weight loss reported by next-of-kin.

### Statistical analysis

We compared characteristics of participants with and without TB multimorbidity, and reported Wilson 95% confidence intervals (CI). Distributions of TB key comorbidities and multimorbidity were assessed using Euler diagrams, stratified by age, sex and site. Associations between demographic and socioeconomic characteristics and TB comorbidities and multimorbidity were investigated using Pearson’s Chi-squared test or Fisher’s exact test for categorical variables, and Wilcoxon’s rank sum test for continuous variables. All analyses were two-sided and statistical significance was considered p < 0·05. Multiple comparisons were corrected for using a false discovery rate approach, reported as q values in tables [11].

### Mortality analysis

The primary outcome measure was all-cause mortality over 15 months from recruitment. Survival curves and mortality rates disaggregated by individual TB key comorbidities and cumulative multimorbidity (0, 1, 2 and ≥3 TB key comorbidities) were estimated using the Kaplan-Meier method. Regression models to assess factors independently associated with increased mortality pre- and post-TB treatment initiation and overall mortality were constructed based upon a predefined set of modelling decisions (Table A in S1 Appendix). Logistic regression was used to model the odds that a participant survived to TB treatment initiation and Cox proportional hazards regression to assess the association of individual comorbidities and TB multimorbidity with mortality from treatment initiation to death or censoring at 15 months. Models were adjusted for age, sex, site, income type, previous TB, and rifampicin resistance and were mutually adjusted. Interaction terms were fitted to assess effect modification due to age, sex and HIV status. Predicted probabilities of death were calculated by Cox proportional hazards based upon combinations of TB key comorbidities and stratified by age, sex and HIV status. Total population attributable fractions of mortality due to individual TB key comorbidities were calculated by the direct method using Cox proportional hazards models including directed acyclic graph-informed adjustment sets. Direct population attributable fractions were calculated using a multivariable Cox proportional hazard model including age, sex and the five TB key comorbidities. A joint population attributable fraction for the TB key comorbidities was estimated using the multivariable Cox model-based counterfactual predictions. Confidence intervals for population attributable fractions were calculated using n = 50 bootstrap replications. Analyses were based on complete case analysis and were conducted using R version 4.3.2 [12 –14].

## Results

### Study population

**Fig 1:**
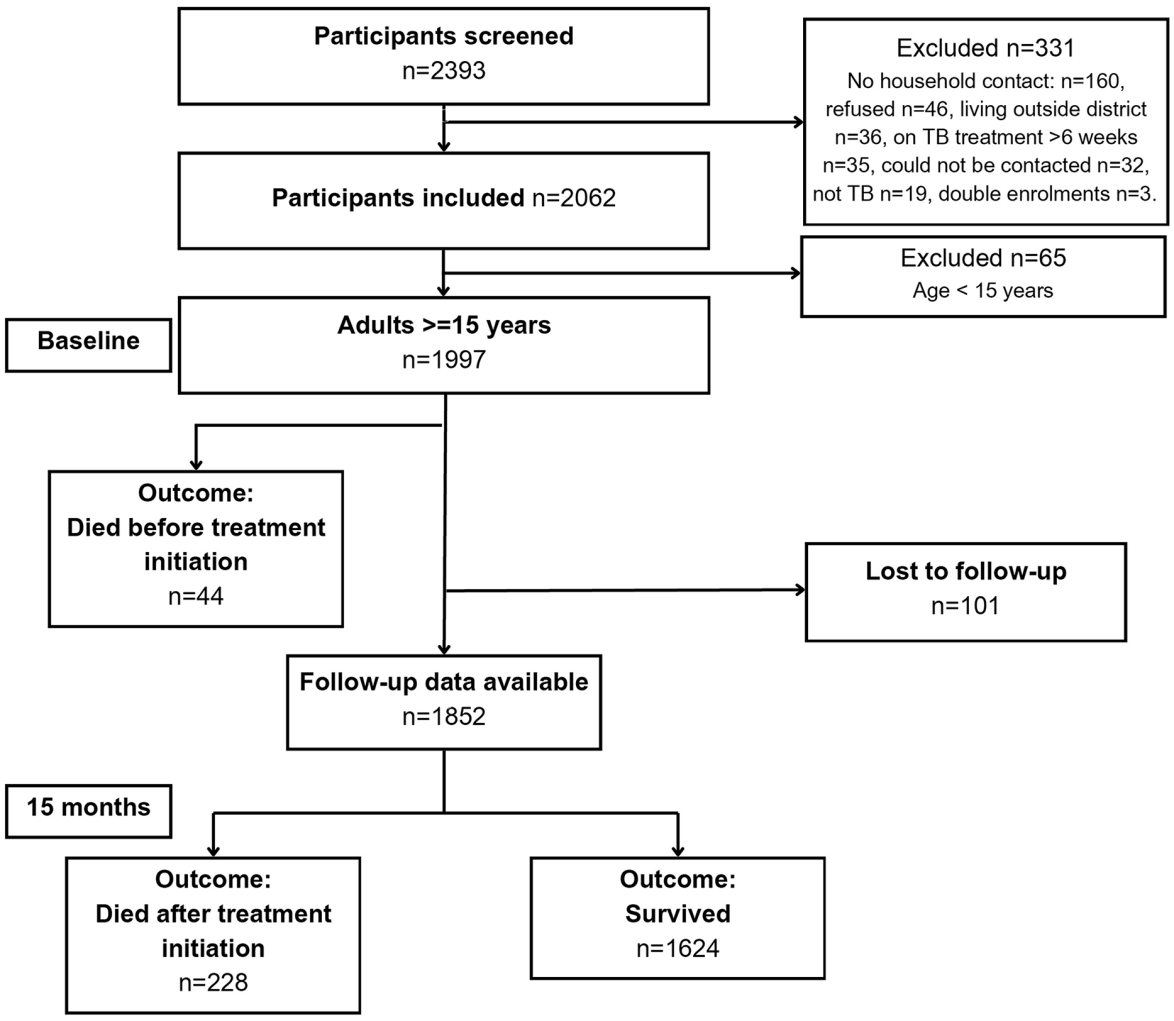
Flow diagram of included patients and outcomes. TB, tuberculosis.

We included 1,997 adult index patients with bacteriologically-confirmed pulmonary TB, of whom 1896 (95%) had outcome data available at 15 months (Fig 1). Their median (interquartile range [IQR]) age was 38 (29–48) years, 1203/1997 (60%) were male, and 1,874 (94%) had a positive sputum Xpert MTB/RIF test, of whom 147 (7%) also had rifampicin resistance on Xpert MTB/RIF (Table 1). In the previous three years, 437/1997 (22%) of participants reported having had a household TB contact, 149/437 (34%) of these contacts were deceased. Most participants, 1132/1997 (57%), had no personal income, 367/1997 (18%) were employed, and 403/1997 (20%) received income through a government grant. Male participants were older, more likely to be employed, and have an income than female participants (Table 1).

**Table 1:** Demographics, socioeconomic context, and TB multimorbidity in adult (≥15 years) participants recruited to the HomeACF study.

Characteristic	Overall, N = 1,997^*1*^	Male, N = 1,203^*1*^	Female, N = 794^*1*^	p-value^*2*^	q-value^*3*^
**Study site**				0·049	0·077
Mangaung	1,045 (52%)	651 (54%)	394 (50%)		
Capricorn	952 (48%)	552 (46%)	400 (50%)		
**Age group (years)**				<0·001	<0·001
<20	91 (4·6%)	38 (3·2%)	53 (6·7%)		
20-29	428 (21%)	228 (19%)	200 (25%)		
30-39	592 (30%)	341 (28%)	251 (32%)		
40-49	425 (21%)	285 (24%)	140 (18%)		
50-59	257 (13%)	173 (14%)	84 (11%)		
≥60	204 (10%)	138 (11%)	66 (8·3%)		
**Employment**				<0·001	<0·001
Currently employed	367 (18%)	254 (21%)	113 (14%)		
Not employed	1,393 (70%)	818 (68%)	575 (72%)		
Student	112 (5·6%)	52 (4·3%)	60 (7·6%)		
Other	125 (6·3%)	79 (6·6%)	46 (5·8%)		
**Income type**				<0·001	<0·001
Salary	351 (18%)	237 (20%)	114 (14%)		
Wage	111 (5·6%)	76 (6·3%)	35 (4·4%)		
Grant	403 (20%)	211 (18%)	192 (24%)		
No income	1,132 (57%)	679 (56%)	453 (57%)		
**Index case income**				<0·001	<0·001
R0	1 (0·1%)	1 (0·2%)	0 (0%)		
R1-R999	210 (24%)	88 (17%)	122 (36%)		
R1000-R4999	547 (63%)	354 (68%)	193 (57%)		
R5000-R9999	79 (9·1%)	59 (11%)	20 (5·9%)		
R10000+	28 (3·2%)	22 (4·2%)	6 (1·8%)		
**Sputum Xpert**				0·7	0·7
Positive	1,874 (94%)	1,133 (94%)	741 (93%)		
Negative	16 (0·8%)	10 (0·8%)	6 (0·8%)		
Not done	107 (5·4%)	60 (5·0%)	47 (5·9%)		
**Rifampicin resistance**				0·5	0·5
Rifampicin resistance detected	147 (8·0%)	88 (7·9%)	59 (8·1%)		
Rifampicin resistance not detected	1,163 (63%)	691 (62%)	472 (64%)		
Not done	535 (29%)	334 (30%)	201 (27%)		
Unknown	152	90	62		
**Sputum smear**				0·7	0·7
Positive	305 (15%)	186 (15%)	119 (15%)		
Negative	60 (3·0%)	39 (3·2%)	21 (2·6%)		
Not done	1,632 (82%)	978 (81%)	654 (82%)		
**Previous TB**	427 (21%)	274 (23%)	153 (19%)	0·061	0·090
**Recent household contact with TB (<3 years)**				<0·001	<0·001
Yes	437 (22%)	207 (17%)	230 (29%)		
No	1,468 (74%)	942 (78%)	526 (66%)		
Don’t know	92 (4·6%)	54 (4·5%)	38 (4·8%)		
**Previous household death from TB**	149 (34%)	56 (27%)	93 (40%)	0·003	0·006
**Current smoker**				<0·001	<0·001
Yes (current smoker)	367 (18%)	348 (29%)	19 (2·4%)		
No (but smoked previously)	484 (24%)	436 (36%)	48 (6·0%)		
Never smoked	1,146 (57%)	419 (35%)	727 (92%)		
**Drinks alcohol**	565 (28%)	470 (39%)	95 (12%)	<0·001	<0·001
**If drink alcohol, units/week**	15 (6, 45)	18 (8, 72)	9 (5, 21)	<0·001	<0·001
Unknown/ Not applicable	1,432	733	699		
**HIV status**				<0·001	<0·001
Positive	1,093 (55%)	581 (48%)	512 (64%)		
Negative	820 (41%)	566 (47%)	254 (32%)		
Unknown	84 (4·2%)	56 (4·7%)	28 (3·5%)		
**Taking antiretroviral therapy**	713 (65%)	358 (62%)	355 (69%)	0·008	0·014
**Diabetes**				0·3	0·4
No Diabetes	1,924 (96%)	1,164 (97%)	760 (96%)		
Diabetes - Insulin injection	10 (0·5%)	7 (0·6%)	3 (0·4%)		
Diabetes - Oral treatment only	59 (3·0%)	31 (2·6%)	28 (3·5%)		
Diabetes - Not treated	4 (0·2%)	1 (<0·1%)	3 (0·4%)		
**Blood glucose (mmol/l)**				0·2	0·3
<7	1,263 (75%)	779 (74%)	484 (77%)		
≥7 ≤ 11·1	374 (22%)	246 (23%)	128 (20%)		
>11·1	41 (2·4%)	23 (2·2%)	18 (2·9%)		
Not measured	319	155	164		
**Blood pressure**				0·5	0·5
Hypotension <90/60	87 (5·2%)	57 (5·5%)	30 (4·8%)		
Normal	1,317 (79%)	826 (79%)	491 (79%)		
Grade 1 hypertension	177 (11%)	113 (11%)	64 (10%)		
Grade 2 hypertension	44 (2·6%)	28 (2·7%)	16 (2·6%)		
Grade 3 hypertension	37 (2·2%)	18 (1·7%)	19 (3·1%)		
Not measured	335	161	174		
**Weight loss (reported)**	1,551 (78%)	942 (78%)	609 (77%)	0·4	0·5
**Body Mass Index**				<0·001	<0·001
<16·5: Severely underweight	384 (20%)	254 (22%)	130 (17%)		
16·5-18·4: Underweight	474 (25%)	323 (28%)	151 (20%)		
18·5-24·9: Healthy range	823 (43%)	492 (43%)	331 (43%)		
25-29·9: Overweight	159 (8·3%)	56 (4·9%)	103 (14%)		
≥30: Obese	70 (3·7%)	24 (2·1%)	46 (6·0%)		
Not measured	87	54	33		

^*1*^ n (%); Median (IQR). ^*2*^ Pearson’s Chi-squared test; Fisher’s exact test; Wilcoxon rank sum test. ^*3*^ False discovery rate correction for multiple testing.

### Prevalence of TB key comorbidities

The prevalence of the five TB key comorbidities were: HIV (54·7% [95% CI 52·5-56·9]); undernutrition (44·9% [43·3-47·7]); alcohol use (28·3% [26·3-30·3]); smoking (18·4% [16·7-20·2]); and diabetes (3·7% [2·9-4·6]) (Table 1). Males were more likely than female participants to be a current smoker (348/1203 (29% [26–32]) vs 19/794 (2·4% [1·5-3·8]), p < 0·001), to drink alcohol (470/1203 (39% [36–42]) vs 95/794 (12% [9·8-14]), p < 0·001) and to have undernutrition (599/1181 (51% [48–54]) vs 295/784 (38% [34–41]), p < 0·001). Females were more likely to have HIV (512/794 (64% [61–68]) vs 581/1203 (48% [45–51]), p < 0·001). Severe undernutrition affected 384/1910 (20%) of participants. Of current smokers, 351/367 (96%) reported being daily smokers, smoking a median (IQR) of 5 (3–10) cigarettes per day. In those drinking alcohol, median weekly consumption was 12 (6–21) units per week.

### TB comorbidity and multimorbidity

TB comorbidity and TB multimorbidity were highly prevalent, impacting 1721/1997 (86·2% [84·6-87·6]) and 948/1997 (47·5% [45.3-49.7]) of participants, respectively. In people living with HIV (PLWH), 390/1093 (35·7% [32·9-38·6]) had one TB key comorbidity, the HIV diagnosis itself, 484/1093 (44·3% [41·3-47·3]) had two TB key comorbidities, and 219/1093 (20·0% [17·7-22·6]) had three or more TB key comorbidities. In HIV negative participants, 383/904 (42·4% [39·1-45·7]) had one TB key comorbidity, 173/904 (19·1% [16·7-21·9]) had two and 72/904 (8·0% [6·3-10·0]) had three or more TB key comorbidities.

**Fig 2:**
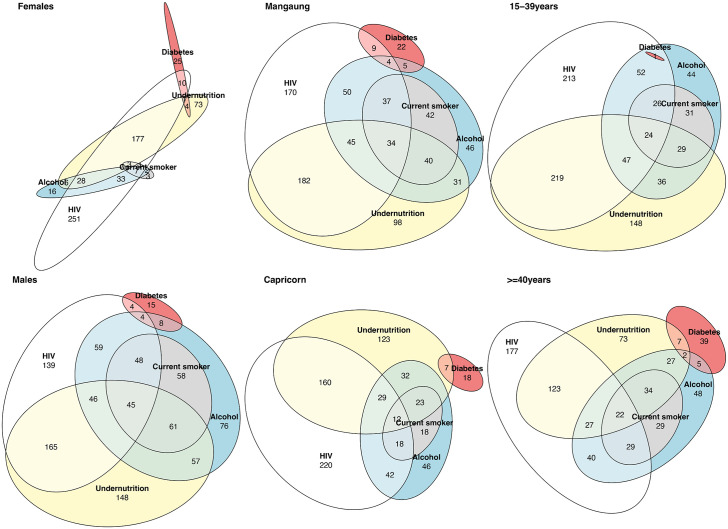
Euler plots, area-proportional venn diagrams, demonstrating the intersection between comorbidities, stratified by sex, site, and age.

**Fig 3:**
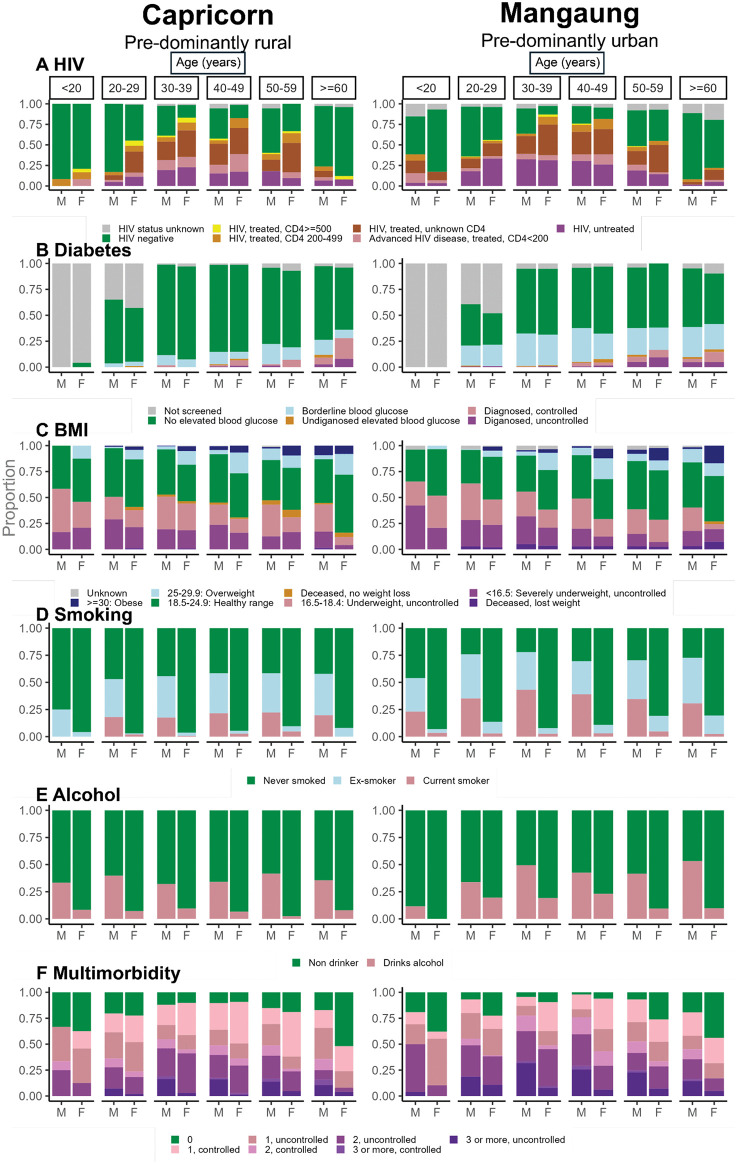
Stacked barchart of diagnosed and undiagnosed TB comorbidities and multimorbidity stratified by age, sex and site. Multimorbidity was considered ‘uncontrolled’ if at least one comorbidity was uncontrolled. Both undernutrition and severe undernutrition were defined as uncontrolled. BMI = body mass index, F = female, M = male. Grouped by age (years).

Excluding smoking and alcohol use, the prevalence of TB comorbidity remained high: 1539/1997 (77·1% [75·1-78·9]). The prevalence and pattern of TB key comorbidities varied by age, sex and urban versus rural setting (Fig 2). TB comorbidity peaked in participants aged 40–49 years (93% [90–95]), was more prevalent in men (male vs female, 90% [88–91] vs 81% [78–84]), and in the urban site (urban vs rural, 88% [86–90] vs 84% [81–86]) (Fig 3, Table B in S1 Appendix).

### Treatment of TB comorbidities

#### HIV.

At the time of TB diagnosis, 713/1093 (65%) PLWH were taking ART. Among HIV negative participants, 716/787 (91%) had been tested for HIV in the last year. Of 84 participants with an unknown HIV status, 61/84 (73%) were in Mangaung and 60/84 (71%) reported never having been tested for HIV.

#### Diabetes.

In patients with pre-existing diabetes, 69/73 (95%) were taking diabetes treatment, 10/69 (14%) on insulin and 59/69 (86%) on oral antidiabetic medication. Random blood glucose was tested in 1426/1478 (96%) of adults aged ≥30 years, of whom 41 (3%) had elevated blood glucose, including 15/41 (37%) not previously diagnosed with diabetes. Participants with diabetes were more likely to be overweight and have high blood pressure, but less likely to be HIV positive (Table C in S1 Appendix).

#### Hypertension.

Pre-existing hypertension was present in 125/1997 (6·3%) of participants, of whom 49/118 (42%) with blood pressure data were hypertensive (Table D and Fig A in S1 Appendix). In those without a pre-existing hypertension diagnosis, 209/1544 (13·5%) were hypertensive.

#### Mortality.

15-month outcome data were available for 1896/1997 (95%) participants, of whom 272/1896 had died, resulting in an overall mortality risk of 14·3% (95% CI: 12·8-16·0). Loss to follow-up was more common in the urban setting and was associated with younger age (Table E in S1 Appendix). Of participants who died, 44/272 deaths (16%) occurred before TB treatment initiation, 41/272 (15%) within one month of TB treatment initiation, a further 101/272 (37%) between one to six months, and 86/272 (32%) after six months. In the 177/257 (69%) deceased PLWH, 114/177 (64%) were reported to be taking ART at the time of death, 25/177 (14%) were not taking ART, and in 38/177 (21%) ART was unknown.

**Fig 4:**
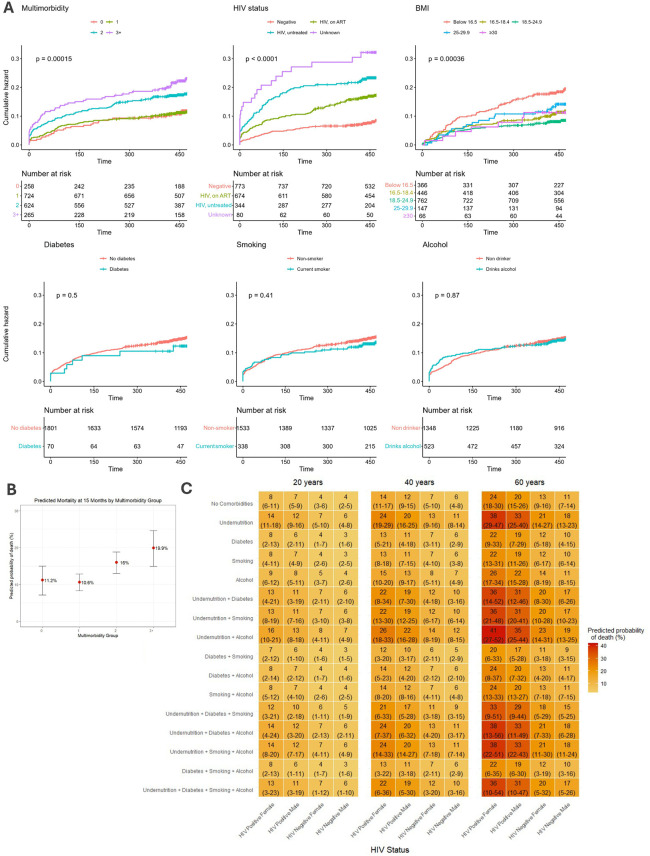
**A)** Kaplan-Meier cumulative hazard survival curves, stratified by multimorbidity and TB key comorbidities including numbers at risk. Time in days. p values = log rank test. **B)** Mortality rates with 95% CI at 15 months by cumulative multimorbidity based upon Kaplan-Meier survival analysis **C)** Multivariate predicted probability of death (95% CI) by Cox proportional hazards stratified by age, sex, combinations TB key comorbidities and HIV status.

Risk of mortality was similar for those with no comorbidities (28/179, 11·2% [7·17-15·0]) and one comorbidity (76/480, 10·6% [8·32-12·8], HR 1·14 [0·72-1·80], p = 0·600), but increased for those with two (99/363, 16·0% [13·0-18·8], HR 1·88 [1·20-2·95], p = 0·006) and three or more (52/144, 19·9% [14·9-24·6], HR 2·58 [1·56-4·24], p < 0·001) TB key comorbidities (Fig 4B, Table L in S1 Appendix). Adjusted for age, sex, site, income type, previous TB, and rifampicin resistance, cumulative TB multimorbidity was associated with increased mortality before and after treatment initiation (Tables H-I and Fig B in S1 Appendix).

Increasing age, HIV positive status, and undernutrition were significantly associated with increased mortality after adjustment for sex, site, income type, previous TB, and rifampicin resistance (Fig 4A, Tables F, J and K in S1 Appendix). The mortality rate was 186/848 (18% [16–20]) in PLWH, 22/59 (27% [18–38]) in those with an unknown HIV status, and 64/717 (8·2% [6·4-10]) in HIV negative participants (p < 0·001). Mortality rates were lowest in those with a BMI in the healthy range (68/705, 8·8% [6·9-11]) and increased incrementally in those with undernutrition (52/400, 12% [8·8-15], HR 1·36 [0·93-2·00], p = 0·12) and severe undernutrition (65/305, 18% [14–22], HR 2·05 [1·43-2·95] p < 0·001). There was evidence of interaction between age and HIV positive status and undernutrition, which indicated that age had a smaller effect on mortality in these groups (Table A in S1 Appendix). There was no interaction between cumulative TB multimorbidity and age or sex, nor individual TB key comorbidities and sex. There was an interaction between an unknown HIV status and alcohol use. In patients with an unknown HIV status, alcohol use was associated with a 5·7 fold increased hazard of death (HR = 5·75 (95% CI: 1·44–23·02), p = 0·0135). A minority, 23/84 (27%), of patients with an unknown HIV status drank alcohol. There were no interactions between undernutrition, diabetes and smoking with HIV status. After adjusting for age and the five TB key comorbidities, there was no sex difference in mortality (Fig 4C).

The total population attributable fraction of mortality due to the five TB key comorbidities was 60.0% (95% CI 49·6 to 69·2%). The population attributable fractions of individual TB key comorbidities were HIV 47·4% (35·1 to 60·5%), undernutrition 24·0% (13·4 to 33·1%), alcohol 0·3% (-6·1 to 8.3%), smoking -1·5% (-7·7 to 4·2%) and diabetes -2.2% (-5·0% to 0·4%) (Table N in S1 Appendix). In the subset of PLWH, the total population attributable fraction of mortality due to ART was -17·5% (-32·3 to -4·8%).

## Discussion

In this prospective cohort study of 1997 adults with bacteriologically-confirmed pulmonary TB in South Africa we found that TB comorbidity and TB multimorbidity were highly prevalent and strongly associated with subsequent mortality. Based upon the study case definitions of the five TB key comorbidities in the WHO Framework for Collaborative Action on TB and Comorbidities, the prevalence of TB comorbidity was 86% and TB multimorbidity was 48%. TB comorbidity was heterogeneous, peaked in middle age, and was higher in men and in the urban setting. The prevalence of four of five TB key comorbidities; HIV, undernutrition, smoking and alcohol use, varied significantly between men and women. Over half of mortality was attributable to TB comorbidity, predominantly due to strong associations between HIV, undernutrition and subsequent mortality. Cumulative TB multimorbidity was associated with incremental mortality risk.

The high prevalence of TB multimorbidity aligns with findings from other high TB burden African countries but contrasts with a retrospective analysis in Brazil, where only 1·14% (454/39,881) of people with TB had multimorbidity [15 ^–^ 17]. However, multimorbidity definitions are heterogeneous, and a significant research gap remains in understanding TB multimorbidity in LMICs, as most studies were historically conducted in high-income countries, often excluding infectious diseases like HIV [18]. In the wider literature, the term “TB multimorbidity” is often used to describe TB with one comorbidity, however, the definitions included in the WHO Framework for Collaborative Action on TB and Comorbidities suggest that multimorbidity should be considered TB with two or more comorbidities [3,19]. To enable meaningful evidence synthesis, future multimorbidity research in TB should include the reporting of findings according to WHO case definitions.

Our finding that over half of mortality is attributable to TB key comorbidities in the study setting supports WHO recommendations for integrated person-centred services [3]. Despite this, a recent survey across 27 high-TB burden countries found that TB providers were not confident in managing comorbidities and, with the exception of HIV, comorbidities are rarely managed within TB services [19]. In South Africa, HIV is reported to be well treated compared to other diseases, including TB and diabetes, and there have been calls for a public health response providing multimorbidity care to tackle convergent infectious and non-communicable disease syndemics [20]. This study partially demonstrated the protective impact of ART, estimating that 1 in 6 deaths in PLWH were averted due to ART in the 15 months following TB. This is an underestimate of the potential benefits of ART, as many PLWH were not on ART, and the group of participants with an ‘unknown’ HIV status who had a high mortality may additionally represent PLWH who were undiagnosed. There is an urgent need to operationalise screening for TB key comorbidities at TB notification, including nutritional interventions, brief interventions for alcohol use, and access to smoking cessation services. Further implementation research and health economic analysis is required to support policy makers and national TB programme managers.

Men in South Africa are more likely to have TB and worse clinical outcomes than women [21]. However, once accounting for age and TB key comorbidities, we did not observe a sex difference in mortality, indicating that TB key comorbidities, among other intersecting factors, may be mediating the relationship between sex and mortality in TB. The greater prevalence of TB multimorbidity in men in our cohort was partly attributable to alcohol use and smoking. We did not find in the overall cohort an increased mortality risk associated with smoking or alcohol use, and some population attributable fractions were negative. The confidence intervals for these estimates crossed zero, and additionally this finding may be related to time-updated misclassification. Smoking and alcohol use have been previously associated with TB relapse and increased prevalence of post-tuberculosis sequelae [22, 23]. It is a limitation that the measurements taken at baseline in this study, including smoking status, alcohol use, nutrition, and blood glucose, may change during illness and treatment, and this may result in misclassification. Further research is required to define the underlying causal pathways, drivers and mediators of morbidity and mortality related to TB multimorbidity.

Our finding that cumulative TB multimorbidity, particularly related to HIV and undernutrition, is strongly associated with deaths before treatment initiation supports WHO recommendations to address health-related risk factors for TB. Multimorbidity is estimated to impact 1 in 4 household members in TB affected households in east and southern Africa, with HIV as the most prevalent comorbidity [24]. It has been suggested that the household may be a unit for intervention, with a role in interrupting the TB poverty cycle [25]. The RATIONS trial in India demonstrated that a nutritional intervention for all people within a TB-affected household resulted in a substantial (39–48%) reduction in TB incidence of contacts [26]. In our cohort, rates of undernutrition were very high, including severe undernutrition affecting 1 in 5 participants, and 24% of mortality was attributable to undernutrition. Worldwide, 98% of the 800 million people who are chronically undernourished live in LMICs where TB is endemic, creating a major risk to public health given the dose-response relationship between undernutrition and TB incidence and death [27, 28]. The existing literature and the evidence from this analysis strongly support the importance of integrated nutritional interventions for TB prevention and care.

This large, prospective study nested within a trial has many strengths, including representation from both urban and rural areas, the large sample size, and a high retention rate of 15-month follow up data (95%). The inclusion of a research-neglected group of individuals with TB who died shortly before recruitment reduces the bias towards underestimating mortality, however, ascertainment of comorbidities is more challenging in this group. We addressed only the five TB key comorbidities defined by WHO and data on other comorbidities known to increase the risk of developing TB disease and/or mortality, such as epilepsy, mental health disorders, chronic kidney disease, and chronic lung disease, were not collected and thus couldn’t be evaluated, but remain an important area of further research [29–31]. We did not include hypertension in the TB multimorbidity definition, which may be an important comorbidity particularly for future cardiac, kidney and cerebrovascular events.

We measured hypertension on three occasions in a single day, however, measurements should be performed on separate occasions for clinical diagnosis. Similarly, we did not have data on ART adherence or viral load and were unable to differentiate between alcohol use and alcohol use disorder. Defining alcohol use as any alcohol consumption was guided by local patterns of alcohol consumption in South Africa, where abstinence from alcohol is common, however, there is a high prevalence of binge drinking and reported denial of alcohol use which may bias mortality estimates [32]. In other contexts, smoking and alcohol use disorder may be considered health-related risk factors rather than comorbidities, however, the WHO Framework emphasises that, “when combined with TB, health-related risk factors are also considered comorbidities, and may increase the risk of poor TB treatment outcomes” [3]. We did not take readings for glycated haemoglobin (HbA1c) which, particularly in the context of acute illness or consumption of anti-TB medication including isoniazid, may be more accurate for diagnosing diabetes than blood glucose. Random blood glucose does not diagnose diabetes, however, misdiagnosis by HbA1c is also reported, particularly early after TB diagnosis [33–35]. The relatively low prevalence of pre-existing diabetes compared to the general population in our cohort is likely due to the young median age. It is unlikely that untreated diabetes has been misclassified as not diabetes in our cohort due to the very low prevalence of hyperglycaemia in screened adults. In deceased participants we did not have height or weight data, and the relationship between TB severity and undernutrition is likely to be bidirectional. Mortality data captured all causes of death, rather than being specific to tuberculosis-related mortality. The direction of association between comorbidities and other factors in this clinical context is not well known, which limits our ability to robustly assess causal interpretation and the mutually adjusted models reported do not infer causality. The intersecting pattern of comorbidities, and the differential impact of individual TB key comorbidities on mortality, indicate that future multimorbidity research should study combinations or clusters of comorbidities, rather than using an additive number of comorbidities. Our findings may not be generalisable to settings with different epidemiology of health-related risk factors for TB in the general population, in particular those with a low HIV prevalence. However, the importance of addressing TB multimorbidity in these settings remains [36].

TB multimorbidity is highly prevalent and strongly associated with subsequent mortality. People with TB should be offered screening and management of comorbidities to reduce risk of death. These strategies will be essential contributors to ending TB globally.

## Supporting information

S1 Appendix Table A: Approach to model development. S1 Appendix. Table B: Demographic and symptomatic factors associated with TB comorbidity. Unadjusted p value by Pearson’s Chi-squared test, Fisher’s exact test and Wilcoxon rank sum test. S1 Appendix. Table C: Demographic factors and comorbidities associated with diabetes. Unadjusted p value by Pearson’s Chi-squared test, Wilcoxon rank sum test and Fisher’s exact test. S1 Appendix. Table D: Blood pressure measurements, stratified by hypertension diagnosis. S1 Appendix Fig A: Stacked bar chart of hypertension diagnoses and blood pressure measurements, stratified by age, sex and site. S1 Appendix Table E: Lost to follow up analysis. Unadjusted p value by Pearson’s Chi-squared test, Wilcoxon rank sum test and Fisher’s exact test. S1 Appendix Table F: Factors associated with mortality. Unadjusted p value by Pearson’s Chi-squared test and Fisher’s exact test. S1 Appendix Table G: Participants recruited after death. Unadjusted p value by Pearson’s Chi-squared test and Fisher’s exact test. S1 Appendix Fig B: Relationship between TB multimorbidity and mortality (models 1 and 2). S1 Appendix Table H: Model 1A: Logistic regression model of multimorbidity and mortality before treatment initiation. Adjusted for age, sex, site, income type, previous TB and rifampicin resistance. S1 Appendix Table I: Model 1B: In those surviving to start treatment, cox proportional hazards model of multimorbidity and survival from point of treatment initiation to follow-up. Adjusted for age, sex, site, income type, previous TB and rifampicin resistance. S1 Appendix Table J: Model 2A: Logistic regression model of TB key comorbidities and mortality before treatment initiation. Adjusted for age, sex, site, income type, previous TB, rifampicin resistance and comorbidities. S1 Appendix Table K: Model 2B: In those surviving to start treatment, cox proportional hazards model of survival from point of treatment initiation to follow-up including comorbidities. Adjusted for age, sex, site, income type, previous TB and rifampicin resistance. S1 Appendix Table L: Model 3: Cox proportional hazards model of overall mortality, including multimorbidity. Adjusted for age, sex, site, income type, previous TB and rifampicin resistance. S1 Appendix Table M: Model 4: Cox proportional hazards model of TB key comorbidities and overall mortality. Adjusted for age, sex, site, income type, previous TB and rifampicin resistance.S1 Appendix Fig C and Table N: Population attributable fractions (PAFs) of mortality due to TB key comorbidities at 15 months. Total PAFs are estimated using directed acyclic graph-informed adjustment sets and Direct PAFs are estimated using a multivariable Cox proportional hazards model including age, sex and the five TB key comorbidities. In the subset of participants living with HIV, the effect of Antiretroviral therapy (ART) was estimated. Confidence intervals were calculated using n = 50 bootstrap replications. S1 Appendix Methods A: Randomisation of household members. S1 Appendix Methods B: Inclusion Criteria for Index Cases.
(DOCX)
