# Supplementary material for: Tuberculosis comorbidities, multimorbidity and mortality amongst adults in South Africa: A prospective cohort study nested in a trial
Source: PLOS Glob Public Health. 2026 Aug 5;6(8):e0006720. doi: 10.1371/journal.pgph.0006720 (PMC13440854; doi:10.1371/journal.pgph.0006720)
Supplement: Supplementary file 1 [file pgph.0006720.s001.docx]

**Supplementary Material**

[S1 Appendix Table A: Approach to model development	2](#_Toc232754787)

[S1 Appendix Table B: Demographic and symptomatic factors associated with TB comorbidity. Unadjusted p value by Pearson’s Chi-squared test, Fisher’s exact test and Wilcoxon rank sum test.	4](#_Toc232754788)

[S1 Appendix Table C: Demographic factors and comorbidities associated with diabetes. Unadjusted p value by Pearson’s Chi-squared test, Wilcoxon rank sum test and Fisher’s exact test.	6](#_Toc232754789)

[S1 Appendix Table D: Blood pressure measurements, stratified by hypertension diagnosis	8](#_Toc232754790)

[S1 Appendix Fig A: Stacked bar chart of hypertension diagnoses and blood pressure measurements, stratified by age, sex and site.	9](#_Toc232754791)

[S1 Appendix Table E: Lost to follow up analysis. Unadjusted p value by Pearson’s Chi-squared test, Wilcoxon rank sum test and Fisher’s exact test.	10](#_Toc232754792)

[S1 Appendix Table F: Factors associated with mortality. Unadjusted p value by Pearson’s Chi-squared test and Fisher’s exact test.	12](#_Toc232754793)

[S1 Appendix Table G: Participants recruited after death. Unadjusted p value by Pearson’s Chi-squared test and Fisher’s exact test.	15](#_Toc232754794)

[S1 Appendix Fig B: Relationship between cumulative TB multimorbidity and mortality (models 1 and 2).	17](#_Toc232754795)

[S1 Appendix Tables: Models 1-4	18](#_Toc232754796)

[S1 Appendix Table H: Model 1A: Logistic regression model of multimorbidity and mortality before treatment initiation. Adjusted for age, sex, site, income type, previous TB and rifampicin resistance.	18](#_Toc232754797)

[S1 Appendix Table I: Model 1B: In those surviving to start treatment, cox proportional hazards model of multimorbidity and survival from point of treatment initiation to follow-up. Adjusted for age, sex, site, income type, previous TB and rifampicin resistance.	19](#_Toc232754798)

[S1 Appendix Table J: Model 2A: Logistic regression model of TB key comorbidities and mortality before treatment initiation. Adjusted for age, sex, site, income type, previous TB, rifampicin resistance and comorbidities.	20](#_Toc232754799)

[S1 Appendix Table K: Model 2B: In those surviving to start treatment, cox proportional hazards model of survival from point of treatment initiation to follow-up including comorbidities. Adjusted for age, sex, site, income type, previous TB and rifampicin resistance.	21](#_Toc232754800)

[S1 Appendix Table L: Model 3: Cox proportional hazards model of overall mortality, including multimorbidity. Adjusted for age, sex, site, income type, previous TB and rifampicin resistance.	22](#_Toc232754801)

[S1 Appendix Table M: Model 4: Cox proportional hazards model of TB key comorbidities and overall mortality. Adjusted for age, sex, site, income type, previous TB and rifampicin resistance.	23](#_Toc232754802)

[S1 Appendix Fig C and Table N: Population attributable fractions (PAFs) of mortality due to TB key comorbidities at 15 months. Total PAFs are estimated using directed acyclic graph-informed adjustment sets and Direct PAFs are estimated using a multivariable Cox proportional hazards model including age, sex and the five TB key comorbidities. In the subset of participants living with HIV, the effect of Antiretroviral therapy (ART) was estimated. Confidence intervals were calculated using n=50 bootstrap replications.	24](#_Toc232754803)

[S1 Appendix Methods A: Randomisation of household members	25](#_Toc232754804)

[S1 Appendix Methods B: Inclusion Criteria for Index Cases	25](#_Toc232754805)

## S1 Appendix Table A: Approach to model development

| **Model** | **Model design** | **Question and interaction** |
| --- | --- | --- |
| **Model 1 - Multimorbidity mortality model** | 1A) Survival to treatment initiation: Logistic regression  Mortality before treatment initiation ~  Demographics: Age + sex + province + income type +  TB factors: Rifampicin resistance + previous TB +  Multimorbidity  1B) Survival after treatment initiation: Cox proportional hazards  Mortality after treatment initiation ~  Demographics: Age + sex + province + income type +  TB factors: Rifampicin resistance + previous TB +  Multimorbidity | Question: What is the relationship between multimorbidity and mortality in adult TB cases before and after treatment initiation?  Interaction assessed in both models: Age, sex - no evidence of interaction |
| **Model 2 - Comorbidity mortality model** | 2A) Survival to treatment initiation: Logistic regression  Mortality before treatment initiation ~  Demographics: Age + sex + province +income type +  TB factors: Rifampicin resistance + previous TB  Comorbidities: Diabetes + HIV + undernutrition + smoking + alcohol  2B) Survival after treatment initiation: Cox proportional hazards  Mortality after treatment initiation ~  Demographics: Age + sex + province + income type +  TB factors: Rifampicin resistance + previous TB +  Comorbidities: Diabetes + HIV + undernutrition + smoking + alcohol | Question: What is the relationship between individual TB key comorbidities and mortality in adult TB cases, before and after treatment initiation?  Interaction assessed for cox proportional hazards model: Age, sex, HIV  There was no evidence of interaction due to sex.  In the Cox proportional hazards model, there was interaction between an unknown HIV status and alcohol consumption. In patients with an unknown HIV status, alcohol use was associated with a 5-fold increased hazard of death (HR = 5. 10 (95% CI: 1.35– 19.25), p=0.016). 23/84 (27%) of patients with unknown HIV status drank alcohol. There were no other interactions due to HIV status.  There was evidence of interaction between age and HIV positive status and age and undernutrition, the coef and exp(coef) were -0.0318, 0.97 (0.95-0.9934) and -0.0230, 0.98 (0.96-0.9985) respectively.  These interactions indicate that even through age still increases mortality risk in those who are HIV positive or have undernutrition, the effect size is smaller. |
| **Model 3** | 3) Overall survival: Cox proportional hazards  Mortality ~  Demographics: Age + sex + province + income type +  TB factors: Rifampicin resistance + previous TB +  Multimorbidity | Question: What is the effect of multimorbidity on mortality in adult TB cases?  This model is used to estimate hazard ratios for multimorbidity on overall survival |
| **Model 4** | 4) Overall survival: Cox proportional hazards  Mortality ~  Demographics: Age + sex + province + income type +  TB factors: Rifampicin resistance + previous TB +  Comorbidities: Diabetes + HIV + undernutrition + smoking + alcohol | Question: What are the effects of individual TB key comorbidities on mortality in adult TB cases?  This model is used to estimate hazard ratios for individual TB key comorbidities on overall survival |

## S1 Appendix Table B: Demographic and symptomatic factors associated with TB comorbidity. Unadjusted p value by Pearson’s Chi-squared test, Fisher’s exact test and Wilcoxon rank sum test.

| **Characteristic** | **Overall**, N = 1,997*^1^* | **No comorbidity**, N = 276*^1^* | **95% CI***^2^* | **TB comorbidity**, N = 1,721*^1^* | **95% CI***^2^* | **p-value***^3^* | **q-value***^4^* |
| --- | --- | --- | --- | --- | --- | --- | --- |
| **Study site** |  |  |  |  |  | 0.005 | 0.011 |
| Mangaung | 1,045 | 123 (12%) | 9.9%, 14% | 922 (88%) | 86%, 90% |  |  |
| Capricorn | 952 | 153 (16%) | 14%, 19% | 799 (84%) | 81%, 86% |  |  |
| **Age group (years)** |  |  |  |  |  | <0.001 | <0.001 |
| <20 | 91 | 29 (32%) | 23%, 43% | 62 (68%) | 57%, 77% |  |  |
| 20-29 | 428 | 72 (17%) | 13%, 21% | 356 (83%) | 79%, 87% |  |  |
| 30-39 | 592 | 53 (9.0%) | 6.8%, 12% | 539 (91%) | 88%, 93% |  |  |
| 40-49 | 425 | 29 (6.8%) | 4.7%, 9.8% | 396 (93%) | 90%, 95% |  |  |
| 50-59 | 257 | 37 (14%) | 10%, 19% | 220 (86%) | 81%, 90% |  |  |
| >=60 | 204 | 56 (27%) | 22%, 34% | 148 (73%) | 66%, 78% |  |  |
| **Sex** |  |  |  |  |  | <0.001 | <0.001 |
| Male | 1,203 | 125 (10%) | 8.8%, 12% | 1,078 (90%) | 88%, 91% |  |  |
| Female | 794 | 151 (19%) | 16%, 22% | 643 (81%) | 78%, 84% |  |  |
| **Employment** |  |  |  |  |  | <0.001 | <0.001 |
| Currently employed | 367 | 46 (13%) | 9.4%, 16% | 321 (87%) | 84%, 91% |  |  |
| Not employed | 1,393 | 170 (12%) | 11%, 14% | 1,223 (88%) | 86%, 89% |  |  |
| Student | 112 | 33 (29%) | 21%, 39% | 79 (71%) | 61%, 79% |  |  |
| Other | 125 | 27 (22%) | 15%, 30% | 98 (78%) | 70%, 85% |  |  |
| **Income type** |  |  |  |  |  | 0.10 | 0.15 |
| Salary | 351 | 42 (12%) | 8.9%, 16% | 309 (88%) | 84%, 91% |  |  |
| Wage | 111 | 21 (19%) | 12%, 28% | 90 (81%) | 72%, 88% |  |  |
| Grant | 403 | 66 (16%) | 13%, 20% | 337 (84%) | 80%, 87% |  |  |
| No income | 1,132 | 147 (13%) | 11%, 15% | 985 (87%) | 85%, 89% |  |  |
| **Rifampicin Resistance** |  |  |  |  |  | 0.5 | 0.5 |
| Rifampicin resistance detected | 147 | 21 (14%) | 9.3%, 21% | 126 (86%) | 79%, 91% |  |  |
| Rifampicin resistance not detected | 1,163 | 164 (14%) | 12%, 16% | 999 (86%) | 84%, 88% |  |  |
| Not done | 535 | 64 (12%) | 9.4%, 15% | 471 (88%) | 85%, 91% |  |  |
| Unknown | 152 | 27 |  | 125 |  |  |  |
| **Previous TB** | 427 | 39 (9.1%) | 6.7%, 12% | 388 (91%) | 88%, 93% | 0.002 | 0.004 |
| **Previous TB household contact** |  |  |  |  |  | 0.073 | 0.14 |
| Yes | 437 | 75 (17%) | 14%, 21% | 362 (83%) | 79%, 86% |  |  |
| No | 1,468 | 189 (13%) | 11%, 15% | 1,279 (87%) | 85%, 89% |  |  |
| Dont know | 92 | 12 (13%) | 7.2%, 22% | 80 (87%) | 78%, 93% |  |  |
| **Cough** | 1,574 | 221 (14%) | 12%, 16% | 1,353 (86%) | 84%, 88% | 0.6 | 0.6 |
| Unknown | 13 | 1 |  | 12 |  |  |  |
| **Cough duration (days)** | 30 (7, 60) | 30 (12, 60) | 39, 50 | 30 (7, 60) | 40, 45 | 0.2 | 0.3 |
| Unknown | 13 | 1 |  | 12 |  |  |  |
| **Productive cough** | 1,166 | 170 (15%) | 13%, 17% | 996 (85%) | 83%, 87% | 0.3 | 0.4 |
| Unknown | 29 | 2 |  | 27 |  |  |  |
| **Productive cough duration (days)** | 14 (0, 30) | 14 (0, 60) | 25, 34 | 14 (0, 30) | 25, 29 | 0.10 | 0.15 |
| Unknown | 29 | 2 |  | 27 |  |  |  |
| **Bloody cough** | 233 (100%) | 37 (16%) | 12%, 21% | 196 (84%) | 79%, 88% | 0.4 | 0.4 |
| Unknown | 17 | 0 |  | 17 |  |  |  |
| **Bloody cough duration (days)** | 0 (0, 0) | 0 (0, 0) | 2.6, 6.5 | 0 (0, 0) | 2.6, 4.1 | 0.3 | 0.4 |
| Unknown | 17 | 0 |  | 17 |  |  |  |
| **Weight loss (self-report)** | 1,551 | 174 (11%) | 9.7%, 13% | 1,377 (89%) | 87%, 90% | <0.001 | <0.001 |
| **Weight loss duration (days)** | 30 (7, 60) | 21 (0, 32) | 28, 54 | 30 (14, 60) | 53, 65 | <0.001 | <0.001 |
| **Night sweats** | 1,118 | 130 (12%) | 9.8%, 14% | 988 (88%) | 86%, 90% | <0.001 | 0.003 |
| Unknown | 23 | 2 |  | 21 |  |  |  |
| **Night sweats duration (days)** | 12 (0, 30) | 0 (0, 30) | 16, 25 | 14 (0, 30) | 25, 29 | <0.001 | 0.002 |
| Unknown | 23 | 2 |  | 21 |  |  |  |
| **Fever** | 802 | 98 (12%) | 10%, 15% | 704 (88%) | 85%, 90% | 0.085 | 0.15 |
| Unknown | 17 | 2 |  | 15 |  |  |  |
| **Fever duration (days)** | 0 (0, 30) | 0 (0, 30) | 12, 20 | 0 (0, 30) | 17, 21 | 0.11 | 0.2 |
| Unknown | 17 | 2 |  | 15 |  |  |  |
| **WHO-4SS** | 1,837 | 242 (13%) | 12%, 15% | 1,595 (87%) | 85%, 88% | 0.005 | 0.011 |
| *^1^* n (%); Median (IQR) | | | | | | | |
| *^2^* CI = Confidence Interval | | | | | | | |
| *^3^* Pearson’s Chi-squared test; Wilcoxon rank sum test | | | | | | | |
| *^4^* False discovery rate correction for multiple testing | | | | | | | |

## S1 Appendix Table C: Demographic factors and comorbidities associated with diabetes. Unadjusted p value by Pearson’s Chi-squared test, Wilcoxon rank sum test and Fisher’s exact test.

| **Characteristic** | **No diabetes**, N = 1,924*^1^* | **Diabetes**, N = 73*^1^* | **p-value***^2^* | **q-value***^3^* |
| --- | --- | --- | --- | --- |
| **Study site** |  |  | 0.4 | 0.4 |
| Mangaung | 1,003 (52%) | 42 (58%) |  |  |
| Capricorn | 921 (48%) | 31 (42%) |  |  |
| **Age group (years)** |  |  |  |  |
| <20 | 91 (4.7%) | 0 (0%) |  |  |
| 20-29 | 426 (22%) | 2 (2.7%) |  |  |
| 30-39 | 587 (31%) | 5 (6.8%) |  |  |
| 40-49 | 408 (21%) | 17 (23%) |  |  |
| 50-59 | 234 (12%) | 23 (32%) |  |  |
| >=60 | 178 (9.3%) | 26 (36%) |  |  |
| **Sex** |  |  | 0.2 | 0.3 |
| Male | 1,164 (60%) | 39 (53%) |  |  |
| Female | 760 (40%) | 34 (47%) |  |  |
| **Employment** |  |  | 0.033 | 0.066 |
| Currently employed | 356 (19%) | 11 (15%) |  |  |
| Not employed | 1,339 (70%) | 54 (74%) |  |  |
| Student | 112 (5.8%) | 0 (0%) |  |  |
| Other | 117 (6.1%) | 8 (11%) |  |  |
| **Income type** |  |  | 0.001 | 0.004 |
| Salary | 340 (18%) | 11 (15%) |  |  |
| Wage | 108 (5.6%) | 3 (4.1%) |  |  |
| Grant | 374 (19%) | 29 (40%) |  |  |
| No income | 1,102 (57%) | 30 (41%) |  |  |
| **Index case income** |  |  | 0.008 | 0.022 |
| R0 | 1 (0.1%) | 0 (0%) |  |  |
| R1-R999 | 207 (25%) | 3 (7.0%) |  |  |
| R1000-R4999 | 516 (63%) | 31 (72%) |  |  |
| R5000-R9999 | 74 (9.0%) | 5 (12%) |  |  |
| R10000+ | 24 (2.9%) | 4 (9.3%) |  |  |
| Unknown | 1,102 | 30 |  |  |
| **Sputum Xpert** |  |  | 0.3 | 0.4 |
| Positive | 1,802 (94%) | 72 (99%) |  |  |
| Negative | 16 (0.8%) | 0 (0%) |  |  |
| Not done | 106 (5.5%) | 1 (1.4%) |  |  |
| **Rifampicin Resistance** |  |  | 0.5 | 0.5 |
| Rifampicin resistance detected | 140 (7.9%) | 7 (9.9%) |  |  |
| Rifampicin resistance not detected | 1,123 (63%) | 40 (56%) |  |  |
| Not done | 511 (29%) | 24 (34%) |  |  |
| Unknown | 150 | 2 |  |  |
| **Sputum smear** |  |  | 0.3 | 0.4 |
| Positive | 292 (15%) | 13 (18%) |  |  |
| Negative | 60 (3.1%) | 0 (0%) |  |  |
| Not done | 1,572 (82%) | 60 (82%) |  |  |
| **Current smoker** |  |  | 0.027 | 0.062 |
| Yes (current smoker) | 359 (19%) | 8 (11%) |  |  |
| No (but smoked previously) | 472 (25%) | 12 (16%) |  |  |
| Never smoked | 1,093 (57%) | 53 (73%) |  |  |
| **Drinks alcohol** | 549 (29%) | 16 (22%) | 0.2 | 0.3 |
| **If drink alcohol, units/week** | 12 (6, 21) | 12 (9, 24) | 0.7 | 0.7 |
| Unknown | 1,488 | 60 |  |  |
| **HIV status (self-report)** |  |  | <0.001 | <0.001 |
| Positive | 1,077 (56%) | 16 (22%) |  |  |
| Negative | 767 (40%) | 53 (73%) |  |  |
| Unknown | 80 (4.2%) | 4 (5.5%) |  |  |
| **Taking antiretroviral therapy** | 700 (65%) | 13 (81%) | 0.2 | 0.3 |
| Unknown | 847 | 57 |  |  |
| **Hypertension (diagnosed)** |  |  | <0.001 | <0.001 |
| No Hypertension | 1,845 (96%) | 26 (36%) |  |  |
| Hypertension - Oral treatment only | 71 (3.7%) | 46 (63%) |  |  |
| Hypertension - Not Treated | 7 (0.4%) | 1 (1.4%) |  |  |
| Unknown | 1 | 0 |  |  |
| **Blood pressure** |  |  | <0.001 | <0.001 |
| Hypotension <90/60 | 288 (18%) | 5 (7.0%) |  |  |
| Normal | 1,072 (67%) | 39 (55%) |  |  |
| Grade 1 hypertension | 164 (10%) | 13 (18%) |  |  |
| Grade 2 hypertension | 36 (2.3%) | 8 (11%) |  |  |
| Grade 3 hypertension | 31 (1.9%) | 6 (8.5%) |  |  |
| Unknown | 333 | 2 |  |  |
| **Body Mass Index** |  |  | <0.001 | <0.001 |
| <16.5: Severely underweight | 382 (21%) | 2 (2.9%) |  |  |
| 16.5-18.4: Underweight | 464 (25%) | 10 (14%) |  |  |
| 18.5-24.9: Healthy range | 790 (43%) | 33 (47%) |  |  |
| 25-29.9: Overweight | 141 (7.7%) | 18 (26%) |  |  |
| >=30: Obese | 63 (3.4%) | 7 (10%) |  |  |
| Unknown | 84 | 3 |  |  |
| *^1^* n (%); Median (IQR) | | | | |
| *^2^* Pearson’s Chi-squared test; Fisher’s exact test; Wilcoxon rank sum test | | | | |
| *^3^* False discovery rate correction for multiple testing | | | | |

## S1 Appendix Table D: Blood pressure measurements, stratified by hypertension diagnosis

| **Characteristic** | **No Hypertension**, N = 1,871*^1^* | **Hypertension**, N = 125*^1^* |
| --- | --- | --- |
| **Measured blood pressure** |  |  |
| Hypotension <90/60 | 282 (18%) | 11 (9.3%) |
| Normal | 1,053 (68%) | 58 (49%) |
| Grade 1 hypertension | 155 (10%) | 22 (19%) |
| Grade 2 hypertension | 30 (1.9%) | 14 (12%) |
| Grade 3 hypertension | 24 (1.6%) | 13 (11%) |
| Unknown | 327 | 7 |
| *^1^* n (%) | | |

## S1 Appendix Fig A: Stacked bar chart of hypertension diagnoses and blood pressure measurements, stratified by age, sex and site.

## S1 Appendix Table E: Lost to follow up analysis. Unadjusted p value by Pearson’s Chi-squared test, Wilcoxon rank sum test and Fisher’s exact test.

| **Characteristic** | **Overall**, N = 1,997*^1^* | **Outcome known**, N = 1,896*^1^* | **Lost to follow-up**, N = 101*^1^* | **p-value***^2^* | **q-value***^3^* |
| --- | --- | --- | --- | --- | --- |
| **Study site** |  |  |  | <0.001 | <0.001 |
| Capricorn | 952 (48%) | 943 (50%) | 9 (8.9%) |  |  |
| Mangaung | 1,045 (52%) | 953 (50%) | 92 (91%) |  |  |
| **Age group (years)** | 38 (29, 48) | 38 (29, 49) | 34 (27, 41) | 0.006 | 0.020 |
| **Sex** |  |  |  | 0.8 | 0.8 |
| Female | 794 (40%) | 755 (40%) | 39 (39%) |  |  |
| Male | 1,203 (60%) | 1,141 (60%) | 62 (61%) |  |  |
| **Income type** |  |  |  | 0.14 | 0.2 |
| Salary | 351 (18%) | 327 (17%) | 24 (24%) |  |  |
| Wage | 111 (5.6%) | 104 (5.5%) | 7 (6.9%) |  |  |
| Grant | 403 (20%) | 390 (21%) | 13 (13%) |  |  |
| No income | 1,132 (57%) | 1,075 (57%) | 57 (56%) |  |  |
| **Sputum Xpert** |  |  |  | 0.014 | 0.036 |
| Positive | 1,874 (94%) | 1,774 (94%) | 100 (99%) |  |  |
| Negative | 16 (0.8%) | 15 (0.8%) | 1 (1.0%) |  |  |
| Not done | 107 (5.4%) | 107 (5.6%) | 0 (0%) |  |  |
| **Rifampicin Resistance** |  |  |  | <0.001 | <0.001 |
| Rifampicin resistance not detected | 1,163 (63%) | 1,129 (65%) | 34 (34%) |  |  |
| Rifampicin resistance detected | 147 (8.0%) | 141 (8.1%) | 6 (6.1%) |  |  |
| Not done | 535 (29%) | 476 (27%) | 59 (60%) |  |  |
| Unknown | 152 | 150 | 2 |  |  |
| **Previous TB** | 427 (21%) | 402 (21%) | 25 (25%) | 0.4 | 0.5 |
| **Sputum smear** |  |  |  | <0.001 | <0.001 |
| Negative | 60 (3.0%) | 60 (3.2%) | 0 (0%) |  |  |
| Positive | 305 (15%) | 303 (16%) | 2 (2.0%) |  |  |
| Not done | 1,632 (82%) | 1,533 (81%) | 99 (98%) |  |  |
| **Current smoker** |  |  |  | 0.050 | 0.11 |
| Non-smoker | 1,630 (82%) | 1,555 (82%) | 75 (74%) |  |  |
| Current smoker | 367 (18%) | 341 (18%) | 26 (26%) |  |  |
| **Drinks alcohol** |  |  |  | 0.2 | 0.3 |
| Abstinent | 1,432 (72%) | 1,365 (72%) | 67 (66%) |  |  |
| Drinks alcohol | 565 (28%) | 531 (28%) | 34 (34%) |  |  |
| **HIV status** |  |  |  | 0.10 | 0.2 |
| Negative | 820 (41%) | 781 (41%) | 39 (39%) |  |  |
| HIV, on ART | 713 (36%) | 683 (36%) | 30 (30%) |  |  |
| HIV, untreated | 380 (19%) | 351 (19%) | 29 (29%) |  |  |
| Unknown | 84 (4.2%) | 81 (4.3%) | 3 (3.0%) |  |  |
| **Diabetes** |  |  |  | 0.2 | 0.3 |
| No diabetes | 1,924 (96%) | 1,824 (96%) | 100 (99%) |  |  |
| Diabetes | 73 (3.7%) | 72 (3.8%) | 1 (1.0%) |  |  |
| **Body Mass Index** |  |  |  | 0.5 | 0.5 |
| <16.5: Severely underweight | 384 (20%) | 370 (20%) | 14 (14%) |  |  |
| 16.5-18.4: Underweight | 474 (25%) | 452 (25%) | 22 (22%) |  |  |
| 18.5-24.9: Healthy range | 823 (43%) | 773 (43%) | 50 (51%) |  |  |
| 25-29.9: Overweight | 159 (8.3%) | 150 (8.3%) | 9 (9.2%) |  |  |
| >=30: Obese | 70 (3.7%) | 67 (3.7%) | 3 (3.1%) |  |  |
| Unknown | 87 | 84 | 3 |  |  |
| *^1^* n (%); Median (IQR) | | | | | |
| *^2^* Pearson’s Chi-squared test; Wilcoxon rank sum test; Fisher’s exact test | | | | | |
| *^3^* False discovery rate correction for multiple testing | | | | | |

## S1 Appendix Table F: Factors associated with mortality. Unadjusted p value by Pearson’s Chi-squared test and Fisher’s exact test.

| **Characteristic** | **Alive**, N = 1,624*^1^* | **95% CI***^2^* | **Deceased**, N = 272*^1^* | **95% CI***^2^* | **p-value***^3^* | **q-value***^4^* |
| --- | --- | --- | --- | --- | --- | --- |
| **Study site** |  |  |  |  | >0.9 | >0.9 |
| Capricorn | 808 (86%) | 83%, 88% | 135 (14%) | 12%, 17% |  |  |
| Mangaung | 816 (86%) | 83%, 88% | 137 (14%) | 12%, 17% |  |  |
| **Age group (years)** |  |  |  |  | <0.001 | 0.001 |
| <20 | 82 (95%) | 88%, 98% | 4 (4.7%) | 1.5%, 12% |  |  |
| 20-29 | 359 (91%) | 87%, 93% | 36 (9.1%) | 6.5%, 13% |  |  |
| 30-39 | 470 (84%) | 81%, 87% | 90 (16%) | 13%, 19% |  |  |
| 40-49 | 349 (85%) | 81%, 88% | 62 (15%) | 12%, 19% |  |  |
| 50-59 | 209 (85%) | 79%, 89% | 38 (15%) | 11%, 21% |  |  |
| >60 | 155 (79%) | 72%, 84% | 42 (21%) | 16%, 28% |  |  |
| **Sex** |  |  |  |  | 0.4 | 0.6 |
| Female | 640 (85%) | 82%, 87% | 115 (15%) | 13%, 18% |  |  |
| Male | 984 (86%) | 84%, 88% | 157 (14%) | 12%, 16% |  |  |
| **Employment** |  |  |  |  | 0.023 | 0.078 |
| Currently employed | 300 (88%) | 84%, 91% | 40 (12%) | 8.6%, 16% |  |  |
| Not employed | 1,133 (85%) | 83%, 87% | 202 (15%) | 13%, 17% |  |  |
| Student | 95 (93%) | 86%, 97% | 7 (6.9%) | 3.0%, 14% |  |  |
| Other | 96 (81%) | 72%, 87% | 23 (19%) | 13%, 28% |  |  |
| **Income type** |  |  |  |  | 0.3 | 0.6 |
| Salary | 284 (87%) | 83%, 90% | 43 (13%) | 9.8%, 17% |  |  |
| Wage | 93 (89%) | 81%, 94% | 11 (11%) | 5.7%, 19% |  |  |
| Grant | 324 (83%) | 79%, 87% | 66 (17%) | 13%, 21% |  |  |
| No income | 923 (86%) | 84%, 88% | 152 (14%) | 12%, 16% |  |  |
| **Sputum Xpert** |  |  |  |  | 0.026 | 0.078 |
| Positive | 1,521 (86%) | 84%, 87% | 253 (14%) | 13%, 16% |  |  |
| Negative | 9 (60%) | 33%, 83% | 6 (40%) | 17%, 67% |  |  |
| Not done | 94 (88%) | 80%, 93% | 13 (12%) | 6.9%, 20% |  |  |
| **Rifampicin Resistance** |  |  |  |  | 0.012 | 0.054 |
| Rifampicin resistance not detected | 965 (85%) | 83%, 87% | 164 (15%) | 13%, 17% |  |  |
| Rifampicin resistance detected | 110 (78%) | 70%, 84% | 31 (22%) | 16%, 30% |  |  |
| Not done | 419 (88%) | 85%, 91% | 57 (12%) | 9.3%, 15% |  |  |
| Unknown | 130 |  | 20 |  |  |  |
| **Previous TB** | 338 (84%) | 80%, 87% | 64 (16%) | 13%, 20% | 0.3 | 0.6 |
| **Sputum smear** |  |  |  |  | 0.079 | 0.2 |
| Negative | 55 (92%) | 81%, 97% | 5 (8.3%) | 3.1%, 19% |  |  |
| Positive | 269 (89%) | 85%, 92% | 34 (11%) | 8.0%, 15% |  |  |
| Not done | 1,300 (85%) | 83%, 87% | 233 (15%) | 13%, 17% |  |  |
| **Current smoker** |  |  |  |  | 0.6 | 0.7 |
| Yes (current smoker) | 298 (87%) | 83%, 91% | 43 (13%) | 9.4%, 17% |  |  |
| No (but smoked previously) | 393 (86%) | 82%, 89% | 65 (14%) | 11%, 18% |  |  |
| Never smoked | 933 (85%) | 83%, 87% | 164 (15%) | 13%, 17% |  |  |
| **Drinks alcohol** |  |  |  |  | 0.6 | 0.7 |
| Abstinent | 1,166 (85%) | 83%, 87% | 199 (15%) | 13%, 17% |  |  |
| Drinks alcohol | 458 (86%) | 83%, 89% | 73 (14%) | 11%, 17% |  |  |
| **HIV status (self-report)** |  |  |  |  | <0.001 | <0.001 |
| Positive | 848 (82%) | 80%, 84% | 186 (18%) | 16%, 20% |  |  |
| Negative | 717 (92%) | 90%, 94% | 64 (8.2%) | 6.4%, 10% |  |  |
| Unknown | 59 (73%) | 62%, 82% | 22 (27%) | 18%, 38% |  |  |
| **Taking antiretroviral therapy** | 572 (84%) | 81%, 86% | 111 (16%) | 14%, 19% | 0.043 | 0.11 |
| Unknown | 776 |  | 86 |  |  |  |
| **Diabetes** |  |  |  |  | 0.4 | 0.6 |
| No diabetes | 1,560 (86%) | 84%, 87% | 264 (14%) | 13%, 16% |  |  |
| Diabetes | 64 (89%) | 79%, 95% | 8 (11%) | 5.3%, 21% |  |  |
| **Blood glucose** |  |  |  |  | 0.7 | 0.7 |
| <7 | 1,047 (87%) | 85%, 89% | 157 (13%) | 11%, 15% |  |  |
| >=7<=11.1 | 310 (88%) | 84%, 91% | 41 (12%) | 8.6%, 16% |  |  |
| >11.1 | 33 (85%) | 69%, 94% | 6 (15%) | 6.4%, 31% |  |  |
| Unknown | 234 |  | 68 |  |  |  |
| **Hypertension** |  |  |  |  | 0.9 | >0.9 |
| No Hypertension | 1,521 (86%) | 84%, 87% | 253 (14%) | 13%, 16% |  |  |
| Hypertension | 103 (85%) | 77%, 91% | 18 (15%) | 9.3%, 23% |  |  |
| Unknown | 0 |  | 1 |  |  |  |
| **Body Mass Index** |  |  |  |  | <0.001 | 0.004 |
| <16.5: Severely underweight | 305 (82%) | 78%, 86% | 65 (18%) | 14%, 22% |  |  |
| 16.5-18.4: Underweight | 400 (88%) | 85%, 91% | 52 (12%) | 8.8%, 15% |  |  |
| 18.5-24.9: Healthy range | 705 (91%) | 89%, 93% | 68 (8.8%) | 6.9%, 11% |  |  |
| 25-29.9: Overweight | 130 (87%) | 80%, 91% | 20 (13%) | 8.5%, 20% |  |  |
| >=30: Obese | 60 (90%) | 79%, 95% | 7 (10%) | 4.7%, 21% |  |  |
| Unknown | 24 |  | 60 |  |  |  |
| **Deceased TB contact** | 118 (86%) | 78%, 91% | 20 (14%) | 9.3%, 22% | 0.4 | 0.6 |
| Unknown | 1,264 |  | 220 |  |  |  |
| *^1^* n (%) | | | | | | |
| *^2^* CI = Confidence Interval | | | | | | |
| *^3^* Pearson’s Chi-squared test; Fisher’s exact test | | | | | | |
| *^4^* False discovery rate correction for multiple testing | | | | | | |

## S1 Appendix Table G: Participants recruited after death. Unadjusted p value by Pearson’s Chi-squared test and Fisher’s exact test.

| **Characteristic** | **Overall**, N = 1,997*^1^* | **Deceased**,  N = 59*^1^* | **Alive**, N = 1,938*^1^* | **p-value***^2^* |
| --- | --- | --- | --- | --- |
| **Study site** |  |  |  | 0.3 |
| Mangaung | 1,045 (52%) | 35 (59%) | 1,010 (52%) |  |
| Capricorn | 952 (48%) | 24 (41%) | 928 (48%) |  |
| **Age group (years)** |  |  |  | 0.2 |
| <20 | 91 (4.6%) | 0 (0%) | 91 (4.7%) |  |
| 20-29 | 428 (21%) | 10 (17%) | 418 (22%) |  |
| 30-39 | 592 (30%) | 20 (34%) | 572 (30%) |  |
| 40-49 | 425 (21%) | 10 (17%) | 415 (21%) |  |
| 50-59 | 257 (13%) | 10 (17%) | 247 (13%) |  |
| >60 | 204 (10%) | 9 (15%) | 195 (10%) |  |
| **Sex** |  |  |  | 0.5 |
| Male | 1,203 (60%) | 33 (56%) | 1,170 (60%) |  |
| Female | 794 (40%) | 26 (44%) | 768 (40%) |  |
| **Employment** |  |  |  | 0.10 |
| Currently employed | 367 (18%) | 9 (15%) | 358 (18%) |  |
| Not employed | 1,393 (70%) | 41 (69%) | 1,352 (70%) |  |
| Student | 112 (5.6%) | 1 (1.7%) | 111 (5.7%) |  |
| Other | 125 (6.3%) | 8 (14%) | 117 (6.0%) |  |
| **Income type** |  |  |  | >0.9 |
| Salary | 351 (18%) | 12 (20%) | 339 (17%) |  |
| Wage | 111 (5.6%) | 3 (5.1%) | 108 (5.6%) |  |
| Grant | 403 (20%) | 12 (20%) | 391 (20%) |  |
| No income | 1,132 (57%) | 32 (54%) | 1,100 (57%) |  |
| **Rifampicin Resistance** |  |  |  | 0.2 |
| Rifampicin resistance detected | 147 (8.0%) | 8 (14%) | 139 (7.8%) |  |
| Rifampicin resistance not detected | 1,163 (63%) | 33 (58%) | 1,130 (63%) |  |
| Not done | 535 (29%) | 16 (28%) | 519 (29%) |  |
| Unknown | 152 | 2 | 150 |  |
| **Previous TB** | 427 (21%) | 12 (20%) | 415 (21%) | 0.8 |
| **Sputum smear** |  |  |  | 0.5 |
| Positive | 305 (15%) | 9 (15%) | 296 (15%) |  |
| Negative | 60 (3.0%) | 0 (0%) | 60 (3.1%) |  |
| Not done | 1,632 (82%) | 50 (85%) | 1,582 (82%) |  |
| **Current smoker** |  |  |  | 0.2 |
| Yes (current smoker) | 367 (18%) | 14 (24%) | 353 (18%) |  |
| No (but smoked previously) | 484 (24%) | 9 (15%) | 475 (25%) |  |
| Never smoked | 1,146 (57%) | 36 (61%) | 1,110 (57%) |  |
| **Drinks alcohol** | 565 (28%) | 24 (41%) | 541 (28%) | 0.032 |
| **HIV status (self-report)** |  |  |  | <0.001 |
| Positive | 1,093 (55%) | 41 (69%) | 1,052 (54%) |  |
| Negative | 820 (41%) | 9 (15%) | 811 (42%) |  |
| Unknown | 84 (4.2%) | 9 (15%) | 75 (3.9%) |  |
| **Taking antiretroviral therapy** | 713 (65%) | 25 (61%) | 688 (65%) | 0.6 |
| Unknown | 904 | 18 | 886 |  |
| **Diabetes** |  |  |  | >0.9 |
| No diabetes | 1,924 (96%) | 57 (97%) | 1,867 (96%) |  |
| Diabetes | 73 (3.7%) | 2 (3.4%) | 71 (3.7%) |  |
| **Hypertension** |  |  |  | 0.4 |
| No Hypertension | 1,871 (94%) | 53 (91%) | 1,818 (94%) |  |
| Hypertension | 125 (6.3%) | 5 (8.6%) | 120 (6.2%) |  |
| Unknown | 1 | 1 | 0 |  |
| **Body Mass Index** |  |  |  | 0.7 |
| <16.5: Severely underweight | 384 (20%) | 1 (25%) | 383 (20%) |  |
| 16.5-18.4: Underweight | 474 (25%) | 0 (0%) | 474 (25%) |  |
| 18.5-24.9: Healthy range | 823 (43%) | 3 (75%) | 820 (43%) |  |
| 25-29.9: Overweight | 159 (8.3%) | 0 (0%) | 159 (8.3%) |  |
| >=30: Obese | 70 (3.7%) | 0 (0%) | 70 (3.7%) |  |
| Unknown | 87 | 55 | 32 |  |
| **Deceased TB contact** | 149 (34%) | 5 (56%) | 144 (34%) | 0.3 |
| Unknown | 1,560 | 50 | 1,510 |  |
| *^1^* n (%) | | | | |
| *^2^* Pearson’s Chi-squared test; Fisher’s exact test | | | | |

## S1 Appendix Fig B: Relationship between cumulative TB multimorbidity and mortality (models 1 and 2).

A) Logistic regression model of mortality before treatment initiation, including age, sex, site, income type, previous TB, rifampicin resistance and multimorbidity as a factor variable. B) In those surviving to start treatment, cox proportional hazards model of survival from point of treatment initiation to follow-up. Adjusted for age, sex, site, income type, previous TB, rifampicin resistance. C) Logistic regression model of mortality before treatment initiation, including age, sex, site, income type, previous TB, rifampicin resistance and comorbidities. D) In those surviving to start treatment, cox proportional hazards model of survival from point of treatment initiation to follow-up including comorbidities. All models are mutually adjusted.

## S1 Appendix Tables: Models 1-4

### S1 Appendix Table H: Model 1A: Logistic regression model of multimorbidity and mortality before treatment initiation. Adjusted for age, sex, site, income type, previous TB and rifampicin resistance.

| **Characteristic** | **OR***^1^* | **95% CI***^1^* | **p-value** |
| --- | --- | --- | --- |
| Age | 1.04 | 1.01, 1.07 | 0.003 |
| Sex |  |  |  |
| Female | — | — |  |
| Male | 0.39 | 0.20, 0.77 | 0.006 |
| Site |  |  |  |
| Capricorn | — | — |  |
| Mangaung | 1.48 | 0.72, 3.10 | 0.3 |
| Income type |  |  |  |
| Salary | — | — |  |
| Wage | 0.25 | 0.01, 1.39 | 0.2 |
| Grant | 0.59 | 0.22, 1.58 | 0.3 |
| No income | 0.67 | 0.31, 1.52 | 0.3 |
| Previous TB |  |  |  |
| No | — | — |  |
| Yes | 0.61 | 0.26, 1.29 | 0.2 |
| Rifampicin resistance |  |  |  |
| Rifampicin resistance not detected | — | — |  |
| Rifampicin resistance detected | 1.91 | 0.67, 4.72 | 0.2 |
| Not done | 0.88 | 0.40, 1.90 | 0.7 |
| Multimorbidity |  |  |  |
| 0 | — | — |  |
| 1 | 4.99 | 0.96, 91.5 | 0.13 |
| 2 | 10.5 | 2.09, 192 | 0.024 |
| 3+ | 19.5 | 3.56, 365 | 0.005 |
| *^1^* OR = Odds Ratio, CI = Confidence Interval | | | |

### S1 Appendix Table I: Model 1B: In those surviving to start treatment, cox proportional hazards model of multimorbidity and survival from point of treatment initiation to follow-up. Adjusted for age, sex, site, income type, previous TB and rifampicin resistance.

| **Characteristic** | **HR***^1^* | **95% CI***^1^* | **p-value** |
| --- | --- | --- | --- |
| Age | 1.02 | 1.01, 1.03 | <0.001 |
| Sex |  |  |  |
| Female | — | — |  |
| Male | 0.78 | 0.58, 1.05 | 0.10 |
| Site |  |  |  |
| Capricorn | — | — |  |
| Mangaung | 0.97 | 0.70, 1.34 | 0.9 |
| Income type |  |  |  |
| Salary | — | — |  |
| Wage | 0.81 | 0.38, 1.71 | 0.6 |
| Grant | 1.00 | 0.62, 1.63 | >0.9 |
| No income | 1.24 | 0.84, 1.85 | 0.3 |
| Previous TB |  |  |  |
| No | — | — |  |
| Yes | 1.12 | 0.81, 1.54 | 0.5 |
| Rifampicin resistance |  |  |  |
| Rifampicin resistance not detected | — | — |  |
| Rifampicin resistance detected | 1.61 | 1.02, 2.54 | 0.041 |
| Not done | 0.79 | 0.54, 1.16 | 0.2 |
| Multimorbidity |  |  |  |
| 0 | — | — |  |
| 1 | 0.98 | 0.61, 1.57 | >0.9 |
| 2 | 1.55 | 0.97, 2.47 | 0.065 |
| 3+ | 1.97 | 1.16, 3.35 | 0.012 |
| *^1^* HR = Hazard Ratio, CI = Confidence Interval | | | |

### S1 Appendix Table J: Model 2A: Logistic regression model of TB key comorbidities and mortality before treatment initiation. Adjusted for age, sex, site, income type, previous TB, rifampicin resistance and comorbidities.

| **Characteristic** | **OR***^1^* | **95% CI***^1^* | **p-value** |
| --- | --- | --- | --- |
| Age | 1.04 | 1.02, 1.07 | 0.002 |
| Sex |  |  |  |
| Female | — | — |  |
| Male | 0.38 | 0.18, 0.79 | 0.011 |
| Site |  |  |  |
| Capricorn | — | — |  |
| Mangaung | 1.06 | 0.49, 2.30 | 0.9 |
| Income type |  |  |  |
| Salary | — | — |  |
| Wage | 0.22 | 0.01, 1.22 | 0.2 |
| Grant | 0.60 | 0.21, 1.65 | 0.3 |
| No income | 0.59 | 0.27, 1.35 | 0.2 |
| Previous TB |  |  |  |
| No | — | — |  |
| Yes | 0.62 | 0.26, 1.34 | 0.3 |
| Rifampicin resistance |  |  |  |
| Rifampicin resistance not detected | — | — |  |
| Rifampicin resistance detected | 2.28 | 0.79, 5.81 | 0.10 |
| Not done | 1.05 | 0.46, 2.37 | >0.9 |
| HIV |  |  |  |
| Negative | — | — |  |
| Positive | 4.04 | 1.68, 11.1 | 0.003 |
| Unknown | 12.2 | 3.75, 40.4 | <0.001 |
| Diabetes |  |  |  |
| No diabetes | — | — |  |
| Diabetes | 1.94 | 0.29, 7.89 | 0.4 |
| Undernutrition |  |  |  |
| No | — | — |  |
| Yes | 3.90 | 1.97, 8.25 | <0.001 |
| Smoking status |  |  |  |
| Non-smoker | — | — |  |
| Current smoker | 1.77 | 0.71, 4.30 | 0.2 |
| Alcohol use |  |  |  |
| Abstinent | — | — |  |
| Drinks alcohol | 1.99 | 0.92, 4.22 | 0.075 |
| *^1^* OR = Odds Ratio, CI = Confidence Interval | | | |

### S1 Appendix Table K: Model 2B: In those surviving to start treatment, cox proportional hazards model of survival from point of treatment initiation to follow-up including comorbidities. Adjusted for age, sex, site, income type, previous TB and rifampicin resistance.

| **Characteristic** | **HR***^1^* | **95% CI***^1^* | **p-value** |
| --- | --- | --- | --- |
| Age | 1.03 | 1.02, 1.04 | <0.001 |
| Sex |  |  |  |
| Female | — | — |  |
| Male | 0.98 | 0.72, 1.34 | >0.9 |
| Site |  |  |  |
| Capricorn | — | — |  |
| Mangaung | 0.92 | 0.66, 1.27 | 0.6 |
| Income type |  |  |  |
| Salary | — | — |  |
| Wage | 0.82 | 0.39, 1.74 | 0.6 |
| Grant | 1.11 | 0.68, 1.81 | 0.7 |
| No income | 1.24 | 0.83, 1.85 | 0.3 |
| Previous TB |  |  |  |
| No | — | — |  |
| Yes | 1.11 | 0.80, 1.53 | 0.5 |
| Rifampicin resistance |  |  |  |
| Rifampicin resistance not detected | — | — |  |
| Rifampicin resistance detected | 1.71 | 1.09, 2.71 | 0.021 |
| Not done | 0.91 | 0.62, 1.33 | 0.6 |
| HIV |  |  |  |
| Negative | — | — |  |
| Positive | 2.45 | 1.74, 3.45 | <0.001 |
| Unknown | 2.89 | 1.55, 5.39 | <0.001 |
| Diabetes |  |  |  |
| No diabetes | — | — |  |
| Diabetes | 0.80 | 0.34, 1.84 | 0.6 |
| Undernutrition |  |  |  |
| No | — | — |  |
| Yes | 1.57 | 1.18, 2.09 | 0.002 |
| Smoking status |  |  |  |
| Non-smoker | — | — |  |
| Current smoker | 0.87 | 0.57, 1.33 | 0.5 |
| Alcohol use |  |  |  |
| Abstinent | — | — |  |
| Drinks alcohol | 0.99 | 0.69, 1.40 | >0.9 |
| *^1^* HR = Hazard Ratio, CI = Confidence Interval | | | |

### S1 Appendix Table L: Model 3: Cox proportional hazards model of overall mortality, including multimorbidity. Adjusted for age, sex, site, income type, previous TB and rifampicin resistance.

| **Characteristic** | **HR***^1^* | **95% CI***^1^* | **p-value** |
| --- | --- | --- | --- |
| Age | 1.03 | 1.02, 1.04 | <0.001 |
| Sex |  |  |  |
| Female | — | — |  |
| Male | 0.69 | 0.53, 0.91 | 0.008 |
| Site |  |  |  |
| Capricorn | — | — |  |
| Mangaung | 1.05 | 0.78, 1.40 | 0.8 |
| Income type |  |  |  |
| Salary | — | — |  |
| Wage | 0.67 | 0.33, 1.34 | 0.3 |
| Grant | 0.90 | 0.59, 1.39 | 0.6 |
| No income | 1.11 | 0.78, 1.57 | 0.6 |
| Previous TB |  |  |  |
| No | — | — |  |
| Yes | 1.01 | 0.75, 1.35 | >0.9 |
| Rifampicin resistance |  |  |  |
| Rifampicin resistance not detected | — | — |  |
| Rifampicin resistance detected | 1.66 | 1.10, 2.49 | 0.016 |
| Not done | 0.81 | 0.58, 1.13 | 0.2 |
| Multimorbidity |  |  |  |
| 0 | — | — |  |
| 1 | 1.14 | 0.72, 1.80 | 0.6 |
| 2 | 1.88 | 1.20, 2.95 | 0.006 |
| 3+ | 2.58 | 1.56, 4.24 | <0.001 |
| *^1^* HR = Hazard Ratio, CI = Confidence Interval | | | |

### S1 Appendix Table M: Model 4: Cox proportional hazards model of TB key comorbidities and overall mortality. Adjusted for age, sex, site, income type, previous TB and rifampicin resistance.

| **Characteristic** | **HR***^1^* | **95% CI***^1^* | **p-value** |
| --- | --- | --- | --- |
| Age | 1.03 | 1.02, 1.04 | <0.001 |
| Sex |  |  |  |
| Female | — | — |  |
| Male | 0.92 | 0.67, 1.27 | 0.6 |
| Site |  |  |  |
| Capricorn | — | — |  |
| Mangaung | 0.95 | 0.68, 1.33 | 0.8 |
| Income type |  |  |  |
| Salary | — | — |  |
| Wage | 0.69 | 0.30, 1.58 | 0.4 |
| Grant | 1.08 | 0.66, 1.78 | 0.8 |
| No income | 1.19 | 0.79, 1.79 | 0.4 |
| Previous TB |  |  |  |
| No | — | — |  |
| Yes | 1.10 | 0.79, 1.53 | 0.6 |
| Rifampicin resistance |  |  |  |
| Rifampicin resistance not detected | — | — |  |
| Rifampicin resistance detected | 1.63 | 1.01, 2.63 | 0.044 |
| Not done | 0.89 | 0.60, 1.32 | 0.6 |
| HIV |  |  |  |
| Negative | — | — |  |
| Positive | 2.27 | 1.60, 3.21 | <0.001 |
| Unknown | 2.36 | 1.21, 4.60 | 0.012 |
| Diabetes |  |  |  |
| No diabetes | — | — |  |
| Diabetes | 0.78 | 0.33, 1.83 | 0.6 |
| Smoking status |  |  |  |
| Non-smoker | — | — |  |
| Current smoker | 0.84 | 0.54, 1.32 | 0.4 |
| Alcohol use |  |  |  |
| Abstinent | — | — |  |
| Drinks alcohol | 0.89 | 0.61, 1.30 | 0.6 |
| BMI |  |  |  |
| 18.5-24.9: Healthy range | — | — |  |
| <16.5: Severely underweight | 2.05 | 1.43, 2.95 | <0.001 |
| 16.5-18.4: Underweight | 1.36 | 0.93, 2.00 | 0.12 |
| 25-29.9: Overweight | 1.33 | 0.77, 2.30 | 0.3 |
| >=30: Obese | 1.06 | 0.48, 2.35 | 0.9 |
| *^1^* HR = Hazard Ratio, CI = Confidence Interval | | | |

## S1 Appendix Fig C and Table N: Population attributable fractions (PAFs) of mortality due to TB key comorbidities at 15 months. Total PAFs are estimated using directed acyclic graph-informed adjustment sets and Direct PAFs are estimated using a multivariable Cox proportional hazards model including age, sex and the five TB key comorbidities. In the subset of participants living with HIV, the effect of Antiretroviral therapy (ART) was estimated. Confidence intervals were calculated using n=50 bootstrap replications.

| **Exposure by TB key comorbidities case definitions** | **Potential mediators** | **Adjustment Set** | **Total PAF (95% CI)**  **Includes only exposure + adjustment set** | **Direct PAF (95% CI)**  **Based on multivariable Cox model** |
| --- | --- | --- | --- | --- |
| HIV  (HIV positive - on ART, HIV positive – untreated, HIV negative (reference) and unknown) | ART  Undernutrition | Age, sex | 0.474  (0.351,0.605) | 0.469  (0.327,0.593) |
| Smoking - current smoker | Diabetes | Age, sex | -0.0146  (-0.0769,0.0424) | -0.00977  (-0.0663,0.0485) |
| Alcohol - alcohol use | Undernutrition | Age, sex | 0.00305  (-0.0617,0.0834) | 0.017  (-0.0556,0.074) |
| Diabetes | None | Age, sex, smoking | -0.022  (-0.0496,0.00367) | -0.000627  (-0.0327,0.0265) |
| Undernutrition - BMI <18·5 kg/m^2^ | None | Age, sex, HIV, alcohol | 0.240  (0.134,0.331) | 0.240  (0.143,0.352) |
| Taking ART  [In HIV positive subset] | Undernutrition | Age, sex | -0.175  (-0.323,-0.0482) | -0.209  (-0.362,0.0307) |

## S1 Appendix Methods A: Randomisation of household members

Household members of TB index cases were randomised to either the intervention or enhanced standard of care arm using computer-generated random numbers, stratified by district and by age. The dwelling within which the household members live was defined as all rooms under a contiguous roofed area linked by doorways or windows through which air can pass. Household members were all individuals who shared airspace for lengthy periods – either slept overnight at least once, or shared at least two meals in the same household as the index case in the 14 days prior to the index case’s diagnosis of TB. For household contacts, in the intervention arm fieldworkers visited homes within 14 days of index case identification, where all household contacts were offered: sputum, tuberculin skin test (TST). TPT for PLHIV, TST >10mm and children <5 years and POC HIV testing. In the standard of care arm, index TB patients were given referral letters for every household member by the recruiting team at the health facility recommending the household contact take the letter to the local clinic and be screened for TB and HIV. The trial could not blind index cases, their households or study field research teams to allocation.

## S1 Appendix Methods B: Inclusion Criteria for Index Cases

1. Any patient (living or recently deceased) diagnosed with TB at any health facility in the study districts in the six weeks prior to recruitment.

a. For index cases with TB older than 7 years of age, laboratory confirmation of pulmonary TB disease is required, evidenced by hard copy lab report of at least one of: a smear positive result for acid fast bacilli (graded as +, ++, or +++); an Xpert MTB/Rif test or other WHO recommended molecular assay positive for M. tuberculosis; a mycobacterial culture positively identified as M tuberculosis; or a histology/cytology report from a biopsy that includes lung tissue that is suggestive of TB disease. LF-LAM may be used to assist in the diagnosis of TB in HIV positive patients with signs and symptoms of TB (pulmonary and/or extrapulmonary) who have a CD4 cell count less than or equal to 100 cells/μL, or HIV positive patients who are seriously ill regardless of CD4 count or with unknown CD4 count. Evidence of a hard copy of recorded LF-LAM result is required.

a. For index cases 7 years and younger, the diagnosis of TB of any organ will be evidenced by – at a minimum- firstly: a recorded diagnosis of TB and its anatomical site in any clinic or hospital record; and secondly the prescription of multidrug TB treatment by a doctor for a child with signs and symptoms suggestive of TB. The presence of signs or symptoms suggestive of TB may be obtained either from the clinical record or from the caregiver.

2. Permanent resident in a household with at least one other person in either the Capricorn Health District or the Matlosana Municipality for a total of at least three months in the six months prior to recruitment.

3. Either the participant, the parent or next of kin of a participant declare they have no plans for relocation of their household to another municipality or health district during the course of follow up.

Exclusion Criteria for Index Cases

1. The research team is unable to obtain informed consent or assent for the following reasons:

a. Participant is unwilling to provide informed consent or assent;

b. The research team is unable to obtain informed consent from the parents of participants younger than 18 years;

c. The research team is unable to obtain consent for participation from the close next-of-kin of adult patients who are too ill to provide their own consent, or have died.

2. Patients with TB who appeared to have developed or contracted TB or while either institutionalized or incarcerated. Similarly, no person incarcerated or institutionalized at the time of diagnosis will be included in the study.

3. If the study team is unable to locate the household of the index case, or if there is no other household member/s able to be recruited at the household, the participant will be withdrawn from the study.
