# Supplementary material for: Tuberculosis comorbidities, multimorbidity and mortality amongst adults in South Africa: A prospective cohort study nested in a trial
Source: PLOS Glob Public Health. 2026 Aug 5;6(8):e0006720. doi: 10.1371/journal.pgph.0006720 (PMC13440854; doi:10.1371/journal.pgph.0006720)
Supplement: Supplementary file 2 [file pgph.0006720.s003.docx]

Dear Prof Hoddinott, Prof Osman, and Reviewers,

**Summary**

Thank you very much for the time taken to review our work; we are grateful for the insightful feedback and suggestions.

We have been able to incorporate the suggested changes and we believe these revisions have enhanced the manuscript. We have addressed each aspect point-by-point below.

**Individual Reviewers’ Comments:**

**Reviewer #1:** Well done for addressing this important, emerging topic in TB research

You have presented a clear analysis and useful findings for this (and similar) settings

Thank you very much for this positive feedback.

There is 1 minor error that requires correction:

Text box 1: the definition of diabetes. >11.1 is referred to as both elevated and uncontrolled, please correct

We are sorry for the lack of clarity on this point, we have clarified the definition in the text as follows:

“In all participants, elevated and borderline random finger-prick blood glucose were defined as >11·1 and 7·0-11·1mmol/L respectively. In those with a diabetes diagnosis, uncontrolled diabetes was defined as random finger-prick blood glucose >11·1 mmol/L.

TB multimorbidity with diabetes included diabetes reported on the standardised questionnaire, or elevated random blood glucose >11·1mmol/L.”

**Reviewer #2:** Thank you for the opportunity to review this manuscript, “Tuberculosis multimorbidity and its impact on mortality amongst adults in South Africa: a prospective cohort study nested in a trial.” The topic—chronic conditions and health-related risk behaviours as contributors to TB mortality—is important. However, I have a major concern regarding the definition and use of the term multimorbidity, which does not align with accepted definitions or with the definition cited in the introduction. Several of the variables included (e.g. any alcohol intake, smoking status) are not chronic conditions and do not meet established criteria for multimorbidity. As currently operationalised, this construct is not consistent with the literature, leading to conceptual, methodological, and interpretative challenges. The term multimorbidity should therefore be reconsidered.

There are also limitations in the modelling approach that restrict causal interpretation and should be clarified or revised. The remainder of the analysis is generally well presented, and the manuscript provides a clear public health message.

A limitation of this review is that none of the referenced tables or figures were available for review; this assessment is therefore based solely on the manuscript text.

Summary: Thank you very much for your detailed and constructive feedback. We have addressed each point below. We apologise that the tables and figures were not available. All figures and tables are included in the current revised submission.

Major comments

Definition of multimorbidity vs comorbidity

1. The manuscript appears to describe TB comorbidity rather than multimorbidity. The WHO framework quoted by the authors explicitly uses “comorbidity” to denote additional conditions alongside TB, consistent with the broader literature. I cannot identify a rationale for redefining this as “multimorbidity,” particularly when the definition diverges from established usage.

Thank you for raising an important point regarding definitions and the uses of the terms “comorbidity” and “multimorbidity”. In the WHO Framework, the following definitions are provided (https://www.who.int/publications/i/item/9789240055056):

***Comorbidity****: A concurrent disease or health condition in a person with tuberculosis (TB).*

***Multimorbidity****: The presence of two or more concurrent diseases or health conditions in a person with TB.*

We agree the Framework uses “comorbidity” to denote a concurrent disease or health condition in an individual with TB. The Framework additionally defines “multimorbidity” as above and describes it as a connected concept, *“the World Health Organization (WHO), in consultation with key stakeholders, has developed a Framework for collaborative action on TB and comorbidities to enhance the response to TB and comorbidities, contributing towards addressing multimorbidity as part of people-centred care” (Executive summary, page 9).*

To ensure consistency with established usage, we have revised the manuscript to include definitions directly quoted from the WHO Framework:

- “Health-related risk factor: A condition, disease or behaviour that increases the likelihood of developing TB.
- Comorbidity: A concurrent disease or health condition in a person with tuberculosis (TB).
- Multimorbidity: The presence of two or more concurrent diseases or health conditions in a person with TB.
- TB Key Comorbidities: The WHO five ‘Key drivers for TB’; diabetes, disorders due to alcohol use, HIV, smoking and undernutrition. These are defined by WHO as both the five key health-related risk factors for TB and the five comorbidities associated with poorer TB treatment outcomes.”

2. The conditions included in the authors’ definition raise concerns. Smoking and alcohol use (as measured here) are health-related risk factors (and the WHO document terms them as such, whilst explaining how they fall into the overall framework, using the term “health related risk factors and comorbidities”) not chronic conditions. The authors have defined alcohol intake and smoking as components of multimorbidity when they do not meet the established definitions of such.

We agree smoking and alcohol are defined as health-related risk factors. In the context of TB, the WHO Framework states that they should also be considered comorbidities, “When combined with TB, health-related risk factors are also considered comorbidities, and may increase the risk of poor TB treatment outcomes.” Therefore, although smoking and alcohol would not be considered comorbidities in the traditional medical model, and therefore components of multimorbidity, when combined with TB, the WHO framework, which forms the backbone of our analysis, states they should be considered comorbidities.

3. Alcohol use disorder—one of the chronic conditions cited in the WHO framework—is not measured. Instead, any alcohol consumption is used. This limitation is acknowledged in the discussion but this divergence of definition should be explicitly stated in the methods.

This divergence is now stated in the methods as below:

“Alcohol use was measured in place of alcohol use disorder.”

This is discussed as a limitation in the discussion.

Overall I believe a more coherent approach would be to frame the paper around WHO-defined TB comorbidities, with the fact that this includes health-related risk factors as well as true comorbidities noted, rather than multimorbidity. Alternatively, alcohol and smoking should be removed from the multimorbidity definition.

We have adjusted the framing to put more emphasis on WHO-defined TB comorbidities. This is now reflected in the title, abstract and introduction. We have retained smoking and alcohol in the analysis as comorbidities as justified above. We have noted this approach in the discussion alongside the justification:

“In other contexts, smoking and alcohol use disorder may be considered health-related risk factors rather than comorbidities, however, the WHO Framework emphasises that, “when combined with TB, health-related risk factors are also considered comorbidities, and may increase the risk of poor TB treatment outcomes”.

Specific comments

• Line 108: The cited WHO document does not discuss multimorbidity; the sentence is therefore misleading.

We have revised the sentence as follows;

“Addressing TB comorbidities is essential to end the TB epidemic and has been recognised as a priority in the landmark WHO Framework for Collaborative Action on TB and Comorbidities.”

• Line 144: A single random glucose does not constitute a diagnostic assessment for diabetes. Please clarify this in methods and limitations. “Elevated glucose” would be more accurate than “diabetes” when based on a single random measurement. Similarly, definitions oh hypertension require measurement on more than one day

We have used the term “elevated glucose” to describe raised blood glucose levels and “diabetes” to describe those with a pre-existing diabetes diagnosis. We have added further clarification to the methods, results, and limitations. This distinction is illustrated in Figure 3, in which patients are stratified by whether they have diabetes, elevated blood glucose, or both. The following limitations have been added to the discussion:

“Random blood glucose does not diagnose diabetes”

“We measured hypertension on three occasions in a single day, however, measurements should be performed on separate occasions for clinical diagnosis.”

• Line 125: Please state whether any reporting standards (e.g. STROBE) were followed.

The following has been added to the methods:

“STROBE guidelines were followed in the reporting of the study.”

• Textbox 1: Smoking and alcohol exposures are likely to change during TB illness. Many people stop smoking due to symptoms and restart during or after treatment. This introduces “time-updated misclassification.” Please report numbers of ex-smokers/the interval between cessation and TB diagnosis; and acknowledge the fact that measurement at baseline may have resulted in misclassification in the limitations. Underweight is also likely to change during treatment, with similar implications; high random glucose may be transient hyperglycaemia, not diabetes.

The number of ex-smokers is included in Table 1 and the association between smoking status and mortality including ex-smokers is reported in Supplementary Table 6. The interval between smoking cessation and TB diagnosis is not known. We have acknowledged this important limitation in the discussion:

“It is a limitation that the measurements taken at baseline in this study, including smoking status, alcohol, nutrition, and blood glucose, may change during illness and treatment, and this may result in misclassification.”

“We did not find an increased mortality risk related to smoking or alcohol, and some population attributable fractions were negative. This finding may be related to time-updated misclassification, the relatively low intensity of smoking and alcohol use in this setting, and inadequate sample size.”

• Textbox 1: “Underweight” (BMI <18.5) is preferable to undernutrition, as it aligns with the WHO definition used.

The definition has been updated as follows, “Undernutrition was defined as body mass index (BMI) category ‘underweight’ <18·5 kg/m^2^. Severe undernutrition was defined as BMI category ‘severely underweight’ <16·5 kg/m^2^.”

• Line 213: The reported diabetes prevalence seems unexpectedly low for a South African cohort with TB. Please comment. The very high diabetes treatment coverage also needs clarification:

o Is the denominator only those with known pre-existing diabetes?

o Could untreated diabetes have been misclassified as not diabetes based on the questionnaire used, explaining both low prevalence and high treatment coverage?

A discussion of these possibilities is warranted.

The denominator is pre-existing diabetes, we have clarified this within the text.

It is possible untreated diabetes could have been misclassified, however, we think this is unlikely as in 1478 patients >30 years in whom we measured random blood glucose, only 41 patients had elevated blood glucose, and 15 of these were not diagnosed with diabetes. This suggests a low prevalence of uncontrolled and untreated diabetes in the population.

A discussion of these possibilities has been added to the manuscript, as follows:

“The relatively low prevalence of pre-existing diabetes compared to the general population in our cohort is likely due to the young median age. It is unlikely that untreated diabetes has been misclassified as not diabetes in our cohort due to the very low prevalence of hyperglycaemia in screened adults.”

• Line 224: Please explicitly state that HIV is excluded and list which comorbidities are included.

This line required clarification and has been revised:

“In people living with HIV (PLWH), 390/1093 (35·7% [32·9-38·6]) had one TB key comorbidity, the HIV diagnosis itself, 484/1093 (44·3% [41·3-47·3]) had two TB key comorbidities, and 219/1093 (20·0% [17·7-22·6]) had three or more TB key comorbidities.”

• Line 257: The choice of mortality intervals requires justification. Subdividing the 1–15-month interval into 1–6 months (on-treatment) and 7–15 months (post-treatment) would more accurately reflect early vs post-treatment mortality patterns, which are important for understanding outcomes in the post-TB period.

Thank you for this suggestion, we have updated the mortality intervals;

“Of participants who died, 44/272 deaths (16%) occurred before TB treatment initiation, 41/272 (15%) within one month of TB treatment initiation, a further 101/272 (37%) between one to six months, and 86/272 (32%) after six months.”

• Line 259: “Not on treatment” is ambiguous. Please clarify whether this refers to:

a) deaths before treatment initiation;

b) deaths after treatment completion;

c) individuals who defaulted from treatment; or

d) a combination of the above.

This represents a combination of the above, however, the underlying data is ambiguous so on review we have elected to remove this statement from the manuscript.

• Line 264: The analysis grouping “one comorbidity” together is problematic. Occasional alcohol intake or infrequent smoking have vastly different expected associations with mortality compared to HIV or severe underweight; yet this analysis considers these of equal importance. Supp figure 2 and the subsequent paragraph confirms there is no association between smoking, alcohol or diabetes and mortality, but there is with HIV and underweight. Thus, the additive number of conditions is fairly uninformative and possibly misleading. I would be more interested to understand how key TB comorbidities cluster (e.g. a Euler or intersection plot) in individuals and “add on” to each other in influencing mortality risk. The mortality association is driven by HIV +/- underweight - but people living with HIV are more likely to smoke and drink alcohol and be underweight (we both know this, and you show this above in the analysis of number of comorbidities stratified by HIV status). Whilst the conclusion that people with more comorbidities are more likely to die is consequently correct – the clustering of these risk factors with HIV in individuals is important and is something this study would be well positioned to explore (see below re causal modelling)..

We apologise that some of the analyses suggested were not available to you during review. In Figure 2, we include Euler plots visualising the intersection between comorbidities, stratified by sex, site and age. In Figure 4C, we report predicted probabilities of death stratified by age, sex, HIV status and combinations of TB key comorbidities. We agree this is more informative than the additive number of comorbidities. We have added the following text to the discussion to reflect this:

“The intersecting pattern of comorbidities, and the differential impact of individual TB key comorbidities on mortality, indicate that future multimorbidity research should study combinations or clusters of comorbidities, rather than using an additive number of comorbidities.”

• Lines 264 & 271: Please provide absolute numbers alongside proportions and CIs.

The absolute numbers have been added to the main text alongside the proportions and CIs.

• Line 271: The modelling strategy appears to use mutually adjusted models, which limits causal interpretation. Select examples:

o Underweight is likely a mediator of HIV’s effect on mortality, not a confounder.

o Comorbidities are not confounders of age–TB relationships (the causal direction is age → comorbidity → TB, not vice versa).

o Drug resistance may be downstream of some exposures (e.g. HIV, alcohol use), making adjustment inappropriate unless the intention is to assess mediation in which case a model with and without this variable would be appropriate.

If causal interpretation is desired, DAG-informed adjustment sets should be used. Otherwise, please clearly justify the predictive modelling approach and its limitations. In the results narrative and tables, the adjustment sets should be clearly stated, they are not at present.

Additional information has been added to the methods and adjustment sets have been added throughout to the narrative text and Table legends. The direction of association between conditions in this clinical context is not well known, which limits our ability to robustly assess causal interpretation. We instead opted to understand which comorbidities were independently associated with mortality after taking account of factors that are known to be associated with mortality.

We did not aim to assess causal inference and we have recognised in the limitations that the approach we have taken does not allow this. We have revised language throughout to ensure causality is not implied.

The below is additionally included in the discussion:

“The direction of association between comorbidities and other factors in this clinical context is not well known, which limits our ability to robustly assess causal interpretation and the mutually adjusted models reported do not infer causality.”

• Line 278: Please report whether an interaction between HIV and underweight was examined.

Yes the interaction between HIV status and the four other TB key comorbidities was examined. The results have been included in the main text as follows;

“There was an interaction between an unknown HIV status and alcohol consumption. In patients with an unknown HIV status, alcohol use was associated with a 5.7 fold increased hazard of death (HR = 5.75 (95% CI: 1.44–23.02), p=0.0135). A minority, 23/84 (27%), of patients with an unknown HIV status drank alcohol. There were no interactions between undernutrition, diabetes and smoking with HIV status.”

• Line 286: Why is a PAF for smoking provided for women but not men? A larger PAF might be expected in men.

The PAF for men was not provided as smoking did not create an increased hazard. The calculated PAF is -3.5%. This has now been added to the main text alongside a discussion of the interpretation including time-updated misclassification.

“We did not find in the overall cohort an increased mortality risk associated with smoking or alcohol use, and some population attributable fractions were negative. This finding may be related to time-updated misclassification, the relatively low intensity of smoking and alcohol use in this setting, and inadequate sample size.”

• Line 283: Please describe PAF calculation methods in the methods section, including:

o how each PAF was computed,

o which effect estimates were used (mutually adjusted or otherwise),

o whether PAFs were summed to generate an overall figure, or this was based on a model of 1 or more condition vs none.

We have now corrected this omission. The methods have been updated as below;

“Population attributable fractions were calculated using Levin’s formula based upon a mutually adjusted Cox proportional hazards model including the five WHO key comorbidities as binary variables. Population attributable fractions for individual comorbidities were summed to generate an overall figure.”

• Line 320: Multimorbidity cannot confound the association between sex (it cannot be upstream of sex) and mortality; mediation is more plausible.

The manuscript has been adjusted to include this revision as below;

“However, once accounting for age and TB multimorbidity, we did not observe a sex difference in mortality, indicating that TB multimorbidity, among other intersecting factors, may be mediating the relationship between sex and mortality in TB.”

• Line 337: PAFs for underweight stratified by HIV status would be informative.

Thank you for this suggestion, we have added these data to the manuscript. Results as below;

“In PLWH, the population attributable fractions were undernutrition 23%, alcohol 4.6%, diabetes 0.8% and smoking -1.2% and in HIV negative participants, undernutrition 20%, diabetes 1.8%, smoking -3.7% and alcohol -3.0%.”

• Line 363: The reference to “reverse causality” is unclear; mortality cannot cause underweight. Please revise.

The text has been revised as follows;

“The relationship between TB severity and undernutrition is likely to be bidirectional”

• The data availability statement says “on reasonable request” – I think this does not conform to Plos GPH standards?

We fully support data sharing, however, public deposition of the data would breach compliance with the requirements of the local research ethics board. The South African Health Research Ethics Council policy is that data sharing requires a fully executed data transfer agreement. We emphasise that no one has ever been declined access to these data. In order to complete this analysis, the study team executed a data transfer agreement between the Perinatal HIV Research Unit and LSTM via this process. The local PI, Prof Martinson, will undertake any requested data transfers agreements to ensure they are expedited. These matters are being discussed with the PLOS GPH team.

Minor comments

• Line 103: Please specify which two conditions are being referred to.

The text has been revised as follows;

“The interaction between TB, diabetes and other comorbidities including HIV is poorly described, as are sex differences.”

• Line 271: Please add “positive” or similar for clarity.

“Positive” has been added for clarity.

• Ensure consistent use of “underweight” (not varying to use undernutrition/underweight/low BMI) throughout.

We have made revisions to ensure consistency.

Thank you very much again for the time taken to review the manuscript and provide detailed, constructive feedback.
