# Supplementary material for: Tuberculosis comorbidities, multimorbidity and mortality amongst adults in South Africa: A prospective cohort study nested in a trial
Source: PLOS Glob Public Health. 2026 Aug 5;6(8):e0006720. doi: 10.1371/journal.pgph.0006720 (PMC13440854; doi:10.1371/journal.pgph.0006720)
Supplement: Supplementary file 3 [file pgph.0006720.s004.docx]

Dear Prof Hoddinott and Reviewers,

**Summary**

Thank you very much for the time taken to review our revised submission, we have made amendments as requested.

We address each aspect point-by-point below.

**Individual Reviewers’ Comments:**

Reviewer #1: Dear Authors

Thank you very much for your responses to the comments
I appreciate the work that you have completed.

1 observation (not a comment for review or response) a strange coincidence the striking visual similarity between Males and Mangaung in the Fig 2 plots - clearly the numbers differ, but interesting that the overlaps appear so similar

Thank you very much.

Reviewer #2: Thank you very much for the opportunity to review the revised manuscript. My previous comments largely have been addressed – and I again emphasise that this is an important, well conducted , piece of research.

A couple of points to clarify – all line numbers refer to the tracked changes version provided.

1. My interpretation of “Multimorbidity: The presence of two or more concurrent diseases or health conditions in a person with TB” is that by defining multimorbidity as two or more conditions IN A PERSON WITH TB, then TB does not itself “count” towards the two or more – it is a pre-requisite. Otherwise, why use this term and provide a different definition to “Comorbidity” (defined as “A concurrent disease or health condition”)?
Reading of the response from the authors, I thought they agreed with me in this (i.e. there is a difference between comorbiditity (1+TB) and multimorbidity (2+TB) – however this is not reflected in the changes in the manuscript.
e.g.
Line 102 “TB multimorbidity, the coexistence of TB and another comorbidity within an individual”
Line 245: “TB multimorbidity was highly prevalent, impacting 1721/1997 (86·2% [84·6-87·6]) of participants” – referring to 1+TB.
Line 253 / this whole paragraph– same use of multimorbidity
Overall, it is now confusing what the authors mean by multimorbidity in the paper as their use of the term (i.e. they are using this as they were in the previous version of the paper) does not to my interpretation conform to the revised definition provided in this new version.

We are sorry that the interpretation of multimorbidity in the manuscript has not been clear. After consideration, the definitions included in the manuscript have been revised as follows. Revisions are underlined;

## **“Multimorbidity definitions**

These study definitions were adapted from the WHO Framework for Collaborative Action on TB and Comorbidities.[^3^](https://www.zotero.org/google-docs/?gkRfJ9)

- Health-related risk factor: A condition, disease or behaviour that increases the likelihood of developing TB.
- Comorbidity: A concurrent disease or health condition in a person with TB.
- Multimorbidity: The presence of two or more concurrent diseases or health conditions in a single person.
- TB Key Comorbidities: The WHO five ‘Key drivers for TB’; diabetes, disorders due to alcohol use, HIV, smoking and undernutrition. These are defined by WHO as both the five key health-related risk factors for TB and the five comorbidities associated with poorer TB treatment outcomes.
- TB Multimorbidity: The presence of two or more concurrent diseases or health conditions in a single person, one of which is TB.”

We hope it is then clear to the reader how the definitions have been operationalised in the analysis. The terminology throughout the manuscript reflects the above definitions. The definition of TB multimorbidity as TB plus ≥1 comorbidity has precedent in the wider literature.

2. Line 104 – suggest “WHO-defined key comorbidities”

This has been updated as suggested.

3. I am still of the view that models adjusted for rifampicin resistance do not make sense in calculation of PAF. PAF, by definition, makes a causal assumption and thus through presentation of PAF in this paper you are making causal inferences – adding a line to the discussion to say that models should not be interpreted causally does not, to me, overcome that. As previously, I believe that construction of causal models (as well as possible) including use of a DAG would result in a much more informative paper and remain concerned about how the PAF data will be interpreted by readers (i.e. causally!). In fact, the authors themselves interpret it in this way in line 332 and repeated in line 341 (“Over half of mortality was attributable to TB multimorbidity”).

This point has been addressed alongside point 4, below.

4. Thank you for clarifying the methods used to calculate PAF. However, to the best of my understanding, Levin’s formula is valid for unadjusted relative risks and does not generally yield unbiased estimates when applied to adjusted hazard ratios from multivariable Cox models. In addition, summing individual PAFs across comorbidities is inappropriate when exposures co-occur, as this double counts overlapping attributable risk and may overestimate the overall preventable fraction. I think the method should include estimating a joint PAF using model-based counterfactual predictions from the multivariable Cox model, which would appropriately account for multimorbidity and avoid overestimation. Finally I would suggest including an uncertainty interval of the PAF in the narrative to make it clear to the reader that the apparently protective smoking PAF has a confidence interval that crosses 1 and therefore this is not statistically unlikely to be due to chance. Review line 362 (“some population attributable fractions were negative”) – I agree time updated misclassification may be important, but this might just be due to chance (it is small and you have not given a CI).

Thank you for the feedback and suggestions. The methodology for calculating population attributable fractions has been revised in line with the suggested approach, including the use of DAG-informed adjustment sets, joint PAF estimation using model-based counterfactual predictions, and the addition of confidence intervals to the PAFs.

The methods section has been updated as follows;

“Total population attributable fractions of mortality due to individual TB key comorbidities were calculated by the Direct method using Cox proportional hazards models including directed acyclic graph informed adjustment sets. Direct population attributable fractions were calculated using a multivariable Cox proportional hazard model including age, sex and the five TB key comorbidities. A joint population attributable fraction for the TB key comorbidities was estimated using multivariable Cox model-based counterfactual predictions. Confidence intervals for population attributable fractions were calculated using n=50 bootstrap replications.”

The following in included in the Supplementary material:

## **“Supplementary Figure and Table 14: Population attributable fractions (PAFs) of mortality due to TB key comorbidities at 15 months. Total PAFs are estimated using directed acyclic graph-informed adjustment sets and Direct PAFs are estimated using a multivariable Cox proportional hazards model including age, sex and the five TB key comorbidities. In the subset of participants living with HIV, the effect of Antiretroviral therapy (ART) was estimated. Confidence intervals were calculated using n=50 bootstrap replications.**

| **Exposure by TB key comorbidities case definitions** | **Potential mediators** | **Adjustment Set** | **Total PAF (95% CI)**  **Includes only exposure + adjustment set** | **Direct PAF (95% CI)**  **Based on multivariable Cox model** |
| --- | --- | --- | --- | --- |
| HIV  (HIV positive - on ART, HIV positive – untreated, HIV negative (reference) and Unknown) | ART  Undernutrition | Age, sex | 0.474  (0.351,0.605) | 0.469  (0.327,0.593) |
| Smoking - current smoker | Diabetes | Age, sex | -0.0146  (-0.0769,0.0424) | -0.00977  (-0.0663,0.0485) |
| Alcohol - alcohol use | Undernutrition | Age, sex | 0.00305  (-0.0617,0.0834) | 0.017  (-0.0556,0.074) |
| Diabetes | None | Age, sex, smoking | -0.022  (-0.0496,0.00367) | -0.000627  (-0.0327,0.0265) |
| Undernutrition - BMI <18·5 kg/m^2^ | None | Age, sex, HIV, alcohol | 0.240  (0.134,0.331) | 0.240  (0.143,0.352) |
| Taking ART  [In HIV positive subset] | Undernutrition | Age, sex | -0.175  (-0.323,-0.0482) | -0.209  (-0.362,0.0307) |

The main text of the manuscript includes the following results;

“The total population attributable fraction of mortality due to the five TB key comorbidities was 60.0% (95% CI 49·6 to 69·2%). The total population attributable fractions of individual TB key comorbidities were HIV 47·4% (35·1 to 60·5%), undernutrition 24·0% (13·4 to 33·1%), alcohol 0·3% (-6·1 to 8.3%), smoking -1·5% (-7·7 to 4·2%) and diabetes -2.2% (-5·0% to 0·4%) (Supplementary Table 14). In the subset of PLWH, the total population attributable fraction of mortality due to ART was -17·5% (-32·3 to -4·8%).”

The discussion has been updated and now includes the following text;

“We did not find in the overall cohort an increased mortality risk associated with smoking or alcohol use, and some population attributable fractions were negative. The confidence intervals for these estimates crossed zero, and additionally this finding may be related to time-updated misclassification.”

5. Figure 2 – what does “uncontrolled undernutrition” mean? How was that defined? What is “uncontrolled” in the multimorbidity plot defined as? Does 2 uncontrolled mean either uncontrolled or both?

Thank you for recognising this omission. The Figure legend has now been updated to clarify the definition of ‘uncontrolled’ multimorbidity and undernutrition as follows:

#### “Figure 3: Stacked barchart of diagnosed and undiagnosed TB multimorbidity stratified by age, sex and site. Multimorbidity was considered ‘uncontrolled’ if at least one comorbidity was uncontrolled. Both undernutrition and severe undernutrition were defined as uncontrolled. BMI = body mass index, F= female, M=male. Grouped by age (years).”

6. Line 259 – suggest “Treatment of TB comorbidities”?

This has been revised as suggested.

7. Line 326 suggest “comorbidity and multimorbidity”

This revision has been included as suggested.

8. Line 328 – You need to make it clear in this sentence )“Based upon the five TB key comorbidities defined in the WHO Framework for Collaborative Action on TB and Comorbidities, the prevalence of TB multimorbidity was 86%) that this is NOT based on the definitions of comorbidities in the WHO framework (due to the definition of alcohol used) and that this is 1 or more condition – i.e. comorbidity and not multimorbidity (as per the above definition).

This has been clarified as follows;

“Based upon the study case definitions of the five TB key comorbidities in the WHO Framework for Collaborative Action on TB and Comorbidities, the prevalence of TB multimorbidity was 86%. This represents participants with TB and at least one TB key comorbidity.”

9. Line 338 - please refer to Stop TB Partnership document words matter and review terminology.

Thank you for highlighting this. We have revised the terminology as follows;

“The high prevalence of TB multimorbidity aligns with findings from other high TB burden African countries but contrasts with a retrospective analysis in Brazil, where only 1·14% (454/39,881) of people with TB had multimorbidity.”

Thank you very much again for the time taken to rereview the manuscript and provide constructive feedback.
