# Supplementary material for: Tuberculosis comorbidities, multimorbidity and mortality amongst adults in South Africa: A prospective cohort study nested in a trial
Source: PLOS Glob Public Health. 2026 Aug 5;6(8):e0006720. doi: 10.1371/journal.pgph.0006720 (PMC13440854; doi:10.1371/journal.pgph.0006720)
Supplement: Supplementary file 4 [file pgph.0006720.s005.docx]

Dear Prof Hoddinott,

Thank you very much for your feedback on the case definitions included in the manuscript which is copied below;

“*One of the reviewers has raised the point about the definitions of co- and multi-morbidity. Their reading of the "WHO framework on tuberculosis and comorbidities" is that "Multimorbidity is the presence of two or more concurrent disease or health conditions in a person with TB." They argue that this means having TB plus at least two other diseases / health conditions. Further, that the WHO definition for "Comorbidity" is having another (one other) concurrent disease or health condition alongside TB. The reviewer notes that you define "multimorbidity as two or more concurrent disease or health conditions in a single person", and "TB multimorbidity as two or more concurrent diseases or health conditions one of which is TB". There is therefore some divergence. What you are calling TB multimorbidity the WHO may be defining as TB comorbidity. And what you are defining as multimorbidity (two diseases / health conditions) concurrently is outside of the WHO definition for multimorbidity (three diseases; TB plus at least two others). The reviewer further notes that prior to the WHO definition, there has been a host of different definitions used in the literature which has caused much confusion and inability to compare / synthesize across the evidence base. Is it at all possible for you to engage this complexity to ensure that your (very important) manuscript does not leave readers similarly confused? Even if this is only in detailed noting of limitations and not a re-doing of any analysis. It may be helpful to reclassify some of what you are labelling as TB multimorbidity as TB co-morbidity as here this is overlap of definitions.*

*Multimorbidity: The presence of two or more concurrent diseases or health conditions in a single person.*

*TB Multimorbidity: The presence of two or more concurrent diseases or health conditions in a single person, one of which is TB.”*

Response: We have revised the manuscript, engaging with the complexity around divergent case definitions and aligning our work as closely as possible to WHO definitions as suggested. The case definitions and terminology included in the manuscript have been adjusted throughout in line with the text below:

“These study definitions are adapted from the WHO Framework for Collaborative Action on TB and Comorbidities.[^3^](https://www.zotero.org/google-docs/?gkRfJ9)

- Health-related risk factor: A condition, disease or behaviour that increases the likelihood of developing TB.
- Comorbidity: A concurrent disease or health condition in a person with TB.
- Multimorbidity: The presence of two or more concurrent diseases or health conditions in a person with TB.
- TB Key Comorbidities: The WHO five ‘Key drivers for TB’; diabetes, disorders due to alcohol use, HIV, smoking and undernutrition. These are defined by WHO as both the five key health-related risk factors for TB and the five comorbidities associated with poorer TB treatment outcomes.

Therefore, the term “TB comorbidity” is used to describe TB with one comorbidity, and the term “TB multimorbidity” is used to describe TB with two or more comorbidities.”

We have additionally included a paragraph to the Introduction outlining the existing challenges with evidence synthesis and the importance of including WHO definitions in reporting. The updated prevalence estimates are included in the manuscript;

“TB comorbidity and TB multimorbidity were highly prevalent, impacting 1721/1997 (86·2% [84·6-87·6]) and 948/1997 (47·5% [45.3-49.7]) of participants, respectively.”

We hope that these revisions provide clarity to readers and increase the utility of the work in policy and practice.
