# Supplementary material for: Tuberculosis comorbidities, multimorbidity and mortality amongst adults in South Africa: A prospective cohort study nested in a trial
Source: PLOS Glob Public Health. 2026 Aug 5;6(8):e0006720. doi: 10.1371/journal.pgph.0006720 (PMC13440854; doi:10.1371/journal.pgph.0006720)
Supplement: Peer review history

# Author comment: Author response to Decision Letter 0

**DOI:** 10.1371/journal.pgph.0006720.r001

**Transfer Alert**

This paper was transferred from another journal. As a result, its full editorial history (including decision letters, peer reviews and author responses) may not be present.

25 Sep 2025

# Peer review: Decision Letter 0

**DOI:** 10.1371/journal.pgph.0006720.r002

**Authors:** Graeme Hoddinott

16 Nov 2025

PGPH-D-25-01800

Tuberculosis multimorbidity and its impact on mortality amongst adults in South Africa: a prospective cohort study nested in a trial

PLOS Global Public Health

Dear Dr. Byrne,

Thank you for submitting your manuscript to PLOS Global Public Health. After careful consideration, we feel that it has merit but does not fully meet PLOS Global Public Health’s publication criteria as it currently stands. Therefore, we invite you to submit a revised version of the manuscript that addresses the points raised during the review process.

==============================

EDITOR:

Thank you for your submission. Overall, I believe that it has a lot of merit. However, I also note the points made by both reviewers. I believe that you can revise to address these, even if that will require some effort. My suggestion is that you do so, unless you feel very strongly that a rebuttal is justified.

==============================

Please submit your revised manuscript by 16 December 2025. If you will need more time than this to complete your revisions, please reply to this message or contact the journal office at globalpubhealth@plos.org. When you're ready to submit your revision, log on to https://www.editorialmanager.com/pgph/ and select the 'Submissions Needing Revision' folder to locate your manuscript file.

Please include the following items when submitting your revised manuscript:

- A rebuttal letter that responds to each point raised by the editor and reviewer(s). You should upload this letter as a separate file labeled 'Response to Reviewers'.
- A marked-up copy of your manuscript that highlights changes made to the original version. You should upload this as a separate file labeled 'Revised Manuscript with Track Changes'.
- An unmarked version of your revised paper without tracked changes. You should upload this as a separate file labeled 'Manuscript'.

Guidelines for resubmitting your figure files are available below the reviewer comments at the end of this letter.

We look forward to receiving your revised manuscript.

Kind regards,

Graeme Hoddinott, Ph.D

Academic Editor

PLOS Global Public Health

Journal Requirements:

1. Please amend your detailed online Financial Disclosure statement. This is published with the article. It must therefore be completed in full sentences and contain the exact wording you wish to be published.

a) State the initials, alongside each funding source, of each author to receive each grant. For example: “This work was supported by the National Institutes of Health (####### to AM; ###### to CJ) and the National Science Foundation (###### to AM).”

b) State what role the funders took in the study. If the funders had no role in your study, please state: “The funders had no role in study design, data collection and analysis, decision to publish, or preparation of the manuscript.”

For more information, please go to our submission guidelines:

https://journals.plos.org/globalpublichealth/s/submission-guidelines#loc-financial-disclosure-statement

2. Please ensure that the funders and grant numbers match between the Financial Disclosure field and the Funding Information tab in your submission form. Note that the funders must be provided in the same order in both places as well.

3. Please update your online Competing Interests statement. If you have no competing interests to declare, please state: “The authors have declared that no competing interests exist.”

4. In the online submission form, you indicated that “Reasonable requests for data will be available from the corresponding author.”.

All PLOS journals now require all data underlying the findings described in their manuscript to be freely available to other researchers, either

a) In a public repository,

b) Within the manuscript itself, or

c) Uploaded as supplementary information.

This policy applies to all data except where public deposition would breach compliance with the protocol approved by your research ethics board. If your data cannot be made publicly available for ethical or legal reasons (e.g., public availability would compromise patient privacy), please explain your reasons by return email and your exemption request will be escalated to the editor for approval. Your exemption request will be handled independently and will not hold up the peer review process, but will need to be resolved should your manuscript be accepted for publication. One of the Editorial team will then be in touch if there are any issues.

5. Please ensure that the Author List in your manuscript file matches the Author List in the online submission form, including initials and order.

6. Please upload copies of your main figures, Figures 1 to 4, which you refer to in your text.

Please provide separate figure files in .tif or .eps format only and ensure that all files are under our size limit of 10MB.

For more information about how to convert your figure files please see our guidelines: https://journals.plos.org/globalpublichealth/s/figures

7. We have noticed that you have uploaded Supporting Information files, but you have not included a list of legends. Please add a full list of legends for your Supporting Information files before or after the references list.

8. If the reviewer comments include a recommendation to cite specific previously published works, please review and evaluate these publications to determine whether they are relevant and should be cited. There is no requirement to cite these works unless the editor has indicated otherwise.

Additional Editor Comments (if provided):

[Note: HTML markup is below. Please do not edit.]

Reviewers' comments:

Reviewer's Responses to Questions

**Comments to the Author**

1. Does this manuscript meet PLOS Global Public Health’s publication criteria? Is the manuscript technically sound, and do the data support the conclusions? The manuscript must describe methodologically and ethically rigorous research with conclusions that are appropriately drawn based on the data presented.

Reviewer #1: Yes

Reviewer #2: Partly

**********

2. Has the statistical analysis been performed appropriately and rigorously?

Reviewer #1: Yes

Reviewer #2: Yes

**********

3. Have the authors made all data underlying the findings in their manuscript fully available (please refer to the Data Availability Statement at the start of the manuscript PDF file)?

The PLOS Data policy requires authors to make all data underlying the findings described in their manuscript fully available without restriction, with rare exception. The data should be provided as part of the manuscript or its supporting information, or deposited to a public repository. For example, in addition to summary statistics, the data points behind means, medians and variance measures should be available. If there are restrictions on publicly sharing data—e.g. participant privacy or use of data from a third party—those must be specified.

Reviewer #1: No

Reviewer #2: No

**********

4. Is the manuscript presented in an intelligible fashion and written in standard English?

PLOS Global Public Health does not copyedit accepted manuscripts, so the language in submitted articles must be clear, correct, and unambiguous. Any typographical or grammatical errors should be corrected at revision, so please note any specific errors here.

Reviewer #1: Yes

Reviewer #2: Yes

**********

5. Review Comments to the Author

Please use the space provided to explain your answers to the questions above. You may also include additional comments for the author, including concerns about dual publication, research ethics, or publication ethics. (Please upload your review as an attachment if it exceeds 20,000 characters)

Reviewer #1: Well done for addressing this important, emerging topic in TB research

You have presented a clear analysis and useful findings for this (and similar) settings

There is 1 minor error that requires correction:

Text box 1: the defintion of diabetes. >11.1 is referred to as both elevated and uncontrolled, please correct

Reviewer #2: Thank you for the opportunity to review this manuscript, “Tuberculosis multimorbidity and its impact on mortality amongst adults in South Africa: a prospective cohort study nested in a trial.” The topic—chronic conditions and health-related risk behaviours as contributors to TB mortality—is important. However, I have a major concern regarding the definition and use of the term multimorbidity, which does not align with accepted definitions or with the definition cited in the introduction. Several of the variables included (e.g. any alcohol intake, smoking status) are not chronic conditions and do not meet established criteria for multimorbidity. As currently operationalised, this construct is not consistent with the literature, leading to conceptual, methodological, and interpretative challenges. The term multimorbidity should therefore be reconsidered.

There are also limitations in the modelling approach that restrict causal interpretation and should be clarified or revised. The remainder of the analysis is generally well presented, and the manuscript provides a clear public health message.

A limitation of this review is that none of the referenced tables or figures were available for review; this assessment is therefore based solely on the manuscript text.

Major comments

Definition of multimorbidity vs comorbidity

1. The manuscript appears to describe TB comorbidity rather than multimorbidity. The WHO framework quoted by the authors explicitly uses “comorbidity” to denote additional conditions alongside TB, consistent with the broader literature. I cannot identify a rationale for redefining this as “multimorbidity,” particularly when the definition diverges from established usage.

2. The conditions included in the authors’ definition raise concerns. Smoking and alcohol use (as measured here) are health-related risk factors (and the WHO document terms them as such, whilst explaining how they fall into the overall framework, using the term “health related risk factors and comorbidities”) not chronic conditions. The authors have defined alcohol intake and smoking as components of multimorbidity when they do not meet the established definitions of such.

3. Alcohol use disorder—one of the chronic conditions cited in the WHO framework—is not measured. Instead, any alcohol consumption is used. This limitation is acknowledged in the discussion but this divergence of definition should be explicitly stated in the methods.

Overall I believe A more coherent approach would be to frame the paper around WHO-defined TB comorbidities, with the fact that this includes health-related risk factors as well as true comorbidities noted, rather than multimorbidity. Alternatively, alcohol and smoking should be removed from the multimorbidity definition.

Specific comments

• Line 108: The cited WHO document does not discuss multimorbidity; the sentence is therefore misleading.

• Line 144: A single random glucose does not constitute a diagnostic assessment for diabetes. Please clarify this in methods and limitations. “Elevated glucose” would be more accurate than “diabetes” when based on a single random measurement. Similarly, definitions oh hypertension require measurement on more than one day

• Line 125: Please state whether any reporting standards (e.g. STROBE) were followed.

• Textbox 1: Smoking and alcohol exposures are likely to change during TB illness. Many people stop smoking due to symptoms and restart during or after treatment. This introduces “time-updated misclassification.” Please report numbers of ex-smokers/the interval between cessation and TB diagnosis; and acknowledge the fact that measurement at baseline may have resulted in misclassification in the limitations. Underweight is also likely to change during treatment, with similar implications; high random glucose may be transient hyperglycaemia, not diabetes.

• Textbox 1: “Underweight” (BMI <18.5) is preferable to undernutrition, as it aligns with the WHO definition used.

• Line 213: The reported diabetes prevalence seems unexpectedly low for a South African cohort with TB. Please comment. The very high diabetes treatment coverage also needs clarification:

o Is the denominator only those with known pre-existing diabetes?

o Could untreated diabetes have been misclassified as not diabetes based on the questionnaire used, explaining both low prevalence and high treatment coverage?

A discussion of these possibilities is warranted.

• Line 224: Please explicitly state that HIV is excluded and list which comorbidities are included.

• Line 257: The choice of mortality intervals requires justification. Subdividing the 1–15-month interval into 1–6 months (on-treatment) and 7–15 months (post-treatment) would more accurately reflect early vs post-treatment mortality patterns, which are important for understanding outcomes in the post-TB period.

• Line 259: “Not on treatment” is ambiguous. Please clarify whether this refers to:

a) deaths before treatment initiation;

b) deaths after treatment completion;

c) individuals who defaulted from treatment; or

d) a combination of the above.

• Line 264: The analysis grouping “one comorbidity” together is problematic. Occasional alcohol intake or infrequent smoking have vastly different expected associations with mortality compared to HIV or severe underweight; yet this analysis considers these of equal importance. Supp figure 2 and the subsequent paragraph confirms there is no association between smoking, alcohol or diabetes and mortality, but there is with HIV and underweight. Thus, the additive number of conditions is fairly uninformative and possibly misleading. I would be more interested to understand how key TB comorbidities cluster (e.g. a Euler or intersection plot) in individuals and “add on” to each other in influencing mortality risk. The mortality association is driven by HIV +/- underweight - but people living with HIV are more likely to smoke and drink alcohol and be underweight (we both know this, and you show this above in the analysis of number of comorbidities stratified by HIV status). Whilst the conclusion that people with more comorbidities are more likely to die is consequently correct – the clustering of these risk factors with HIV in individuals is important and is something this study would be well positioned to explore (see below re causal modelling)..

• Lines 264 & 271: Please provide absolute numbers alongside proportions and CIs.

• Line 271: The modelling strategy appears to use mutually adjusted models, which limits causal interpretation. Select examples:

o Underweight is likely a mediator of HIV’s effect on mortality, not a confounder.

o Comorbidities are not confounders of age–TB relationships (the causal direction is age → comorbidity → TB, not vice versa).

o Drug resistance may be downstream of some exposures (e.g. HIV, alcohol use), making adjustment inappropriate unless the intention is to assess mediation in which case a model with and without this variable would be appropriate.

If causal interpretation is desired, DAG-informed adjustment sets should be used. Otherwise, please clearly justify the predictive modelling approach and its limitations. In the results narrative and tables, the adjustment sets should be clearly stated, they are not at present.

• Line 278: Please report whether an interaction between HIV and underweight was examined.

• Line 286: Why is a PAF for smoking provided for women but not men? A larger PAF might be expected in men.

• Line 283: Please describe PAF calculation methods in the methods section, including:

o how each PAF was computed,

o which effect estimates were used (mutually adjusted or otherwise),

o whether PAFs were summed to generate an overall figure, or this was based on a model of 1 or more condition vs none.

• Line 320: Multimorbidity cannot confound the association between sex (it cannot be upstream of sex) and mortality; mediation is more plausible.

• Line 337: PAFs for underweight stratified by HIV status would be informative.

• Line 363: The reference to “reverse causality” is unclear; mortality cannot cause underweight. Please revise.

• The data availability statement says “on reasonable request” – I think this does not conform to Plos GPH standards?

Minor comments

• Line 103: Please specify which two conditions are being referred to.

• Line 271: Please add “positive” or similar for clarity.

• Ensure consistent use of “underweight” (not varying to use undernutrition/underweight/low BMI) throughout.

**********

6. PLOS authors have the option to publish the peer review history of their article (what does this mean?). If published, this will include your full peer review and any attached files.

**Do you want your identity to be public for this peer review?**  If you choose “no”, your identity will remain anonymous but your review may still be made public.

For information about this choice, including consent withdrawal, please see our Privacy Policy.

Reviewer #1: **Yes:**  Muhammad Osman

Reviewer #2: No

**********

[NOTE: If reviewer comments were submitted as an attachment file, they will be attached to this email and accessible via the submission site. Please log into your account, locate the manuscript record, and check for the action link "View Attachments". If this link does not appear, there are no attachment files.]

-->Figure Resubmissions:

While revising your submission, we strongly recommend that you use PLOS’s NAAS tool (https://ngplosjournals.pagemajik.ai/artanalysis) to test your figure files. NAAS can convert your figure files to the TIFF file type and meet basic requirements (such as print size, resolution), or provide you with a report on issues that do not meet our requirements and that NAAS cannot fix.

After uploading your figures to PLOS’s NAAS tool - https://ngplosjournals.pagemajik.ai/artanalysis, NAAS will process the files provided and display the results in the "Uploaded Files" section of the page as the processing is complete. If the uploaded figures meet our requirements (or NAAS is able to fix the files to meet our requirements), the figure will be marked as "fixed" above. If NAAS is unable to fix the files, a red "failed" label will appear above. When NAAS has confirmed that the figure files meet our requirements, please download the file via the download option, and include these NAAS processed figure files when submitting your revised manuscript.

# Author comment: Author response to Decision Letter 1

**DOI:** 10.1371/journal.pgph.0006720.r003

19 Feb 2026

Attachment Submitted filename: TBMM PLOS GPH Response to Reviewers_151225.docx

# Peer review: Decision Letter 1

**DOI:** 10.1371/journal.pgph.0006720.r004

**Authors:** Graeme Hoddinott, Graeme Hoddinott

2 Mar 2026

PGPH-D-25-01800R1

Tuberculosis comorbidities, multimorbidity and mortality amongst adults in South Africa: a prospective cohort study nested in a trial

PLOS Global Public Health

Dear Dr. Wingfield,

Thank you for submitting your manuscript to PLOS Global Public Health. After careful consideration, we feel that it has merit but does not fully meet PLOS Global Public Health’s publication criteria as it currently stands. Therefore, we invite you to submit a revised version of the manuscript that addresses the points raised during the review process.

EDITOR:

Overall, please note that both reviewers have commended the quality of the paper and revisions already concluded. Please note that one reviewer still asks for a substantial revision to address the method / PAF.

Please submit your revised manuscript by Apr 16 2026 11:59PM. If you will need more time than this to complete your revisions, please reply to this message or contact the journal office at globalpubhealth@plos.org. When you're ready to submit your revision, log on to https://www.editorialmanager.com/pgph/ and select the 'Submissions Needing Revision' folder to locate your manuscript file.

Please include the following items when submitting your revised manuscript:

- A letter that responds to each point raised by the editor and reviewer(s). You should upload this letter as a separate file labeled 'Response to Reviewers'.
- A marked-up copy of your manuscript that highlights changes made to the original version. You should upload this as a separate file labeled 'Revised Manuscript with Track Changes'.
- An unmarked version of your revised paper without tracked changes. You should upload this as a separate file labeled 'Manuscript'.

Guidelines for resubmitting your figure files are available below the reviewer comments at the end of this letter.

We look forward to receiving your revised manuscript.

Kind regards,

Graeme Hoddinott, Ph.D

Academic Editor

PLOS Global Public Health

Journal Requirements:

If the reviewer comments include a recommendation to cite specific previously published works, please review and evaluate these publications to determine whether they are relevant and should be cited. There is no requirement to cite these works unless the editor has indicated otherwise.

Additional Editor Comments (if provided):

[Note: HTML markup is below. Please do not edit.]

Reviewers' comments:

Reviewer's Responses to Questions

**Comments to the Author**

1. If the authors have adequately addressed your comments raised in a previous round of review and you feel that this manuscript is now acceptable for publication, you may indicate that here to bypass the “Comments to the Author” section, enter your conflict of interest statement in the “Confidential to Editor” section, and submit your "Accept" recommendation.

Reviewer #1: All comments have been addressed

Reviewer #2: (No Response)

**********

2. Does this manuscript meet PLOS Global Public Health’s publication criteria? Is the manuscript technically sound, and do the data support the conclusions? The manuscript must describe methodologically and ethically rigorous research with conclusions that are appropriately drawn based on the data presented.

Reviewer #1: Yes

Reviewer #2: Yes

**********

3. Has the statistical analysis been performed appropriately and rigorously?

Reviewer #1: Yes

Reviewer #2: No

**********

4. Have the authors made all data underlying the findings in their manuscript fully available (please refer to the Data Availability Statement at the start of the manuscript PDF file)?

The PLOS Data policy requires authors to make all data underlying the findings described in their manuscript fully available without restriction, with rare exception. The data should be provided as part of the manuscript or its supporting information, or deposited to a public repository. For example, in addition to summary statistics, the data points behind means, medians and variance measures should be available. If there are restrictions on publicly sharing data—e.g. participant privacy or use of data from a third party—those must be specified.

Reviewer #1: Yes

Reviewer #2: No

**********

5. Is the manuscript presented in an intelligible fashion and written in standard English?

PLOS Global Public Health does not copyedit accepted manuscripts, so the language in submitted articles must be clear, correct, and unambiguous. Any typographical or grammatical errors should be corrected at revision, so please note any specific errors here.

Reviewer #1: Yes

Reviewer #2: Yes

**********

6. Review Comments to the Author

Please use the space provided to explain your answers to the questions above. You may also include additional comments for the author, including concerns about dual publication, research ethics, or publication ethics. (Please upload your review as an attachment if it exceeds 20,000 characters)

Reviewer #1: Dear Authors

Thank you very much for your responses to the comments

I appreciate the work that you have completed.

1 observation (not a comment for review or response) a strange coincidence the striking visual similarity between Males and Mangaung in the Fig 2 plots - clearly the numbers differ, but interesting that the overlaps appear so similar

Reviewer #2: Thank you very much for the opportunity to review the revised manuscript. My previous comments largely have been addressed – and I again emphasise that this is an important, well conducted , piece of research.

A couple of points to clarify – all line numbers refer to the tracked changes version provided.

1. My interpretation of “Multimorbidity: The presence of two or more concurrent diseases or health conditions in a person with TB” is that by defining multimorbidity as two or more conditions IN A PERSON WITH TB, then TB does not itself “count” towards the two or more – it is a pre-requisite. Otherwise, why use this term and provide a different definition to “Comorbidity” (defined as “A concurrent disease or health condition”)?

Reading of the response from the authors, I thought they agreed with me in this (i.e. there is a difference between comorbiditity (1+TB) and multimorbidity (2+TB) – however this is not reflected in the changes in the manuscript.

e.g.

Line 102 “TB multimorbidity, the coexistence of TB and another comorbidity within an individual”

Line 245: “TB multimorbidity was highly prevalent, impacting 1721/1997 (86·2% [84·6-87·6]) of participants” – referring to 1+TB.

Line 253 / this whole paragraph– same use of multimorbidity

Overall, it is now confusing what the authors mean by multimorbidity in the paper as their use of the term (i.e. they are using this as they were in the previous version of the paper) does not to my interpretation conform to the revised definition provided in this new version.

2. Line 104 – suggest “WHO-defined key comorbidities”

3. I am still of the view that models adjusted for rifampicin resistance do not make sense in calculation of PAF. PAF, by definition, makes a causal assumption and thus through presentation of PAF in this paper you are making causal inferences – adding a line to the discussion to say that models should not be interpreted causally does not, to me, overcome that. As previously, I believe that construction of causal models (as well as possible) including use of a DAG would result in a much more informative paper and remain concerned about how the PAF data will be interpreted by readers (i.e. causally!). In fact, the authors themselves interpret it in this way in line 332 and repeated in line 341 (“Over half of mortality was attributable to TB multimorbidity”).

4. Thank you for clarifying the methods used to calculate PAF. However, to the best of my understanding, Levin’s formula is valid for unadjusted relative risks and does not generally yield unbiased estimates when applied to adjusted hazard ratios from multivariable Cox models. In addition, summing individual PAFs across comorbidities is inappropriate when exposures co-occur, as this double counts overlapping attributable risk and may overestimate the overall preventable fraction. I think the method should include estimating a joint PAF using model-based counterfactual predictions from the multivariable Cox model, which would appropriately account for multimorbidity and avoid overestimation. Finally I would suggest including an uncertainty interval of the PAF in the narrative to make it clear to the reader that the apparently protective smoking PAF has a confidence interval that crosses 1 and therefore this is not statistically unlikely to be due to chance. Review line 362 (“some population attributable fractions were negative”) – I agree time updated misclassification may be important, but this might just be due to chance (it is small and you have not given a CI).

5. Figure 2 – what does “uncontrolled undernutrition” mean? How was that defined? What is “uncontrolled” in the multimorbidity plot defined as? Does 2 uncontrolled mean either uncontrolled or both?

6. Line 259 – suggest “Treatment of TB comorbidities”?

7. Line 326 suggest “comorbidity and multimorbidity”

8. Line 328 – You need to make it clear in this sentence )“Based upon the five TB key comorbidities defined in the WHO Framework for Collaborative Action on TB and Comorbidities, the prevalence of TB multimorbidity was 86%) that this is NOT based on the definitions of comorbidities in the WHO framework (due to the definition of alcohol used) and that this is 1 or more condition – i.e. comorbidity and not multimorbidity (as per the above definition).

9. Line 338 - please refer to Stop TB Partnership document words matter and review terminology.

**********

7. PLOS authors have the option to publish the peer review history of their article (what does this mean?). If published, this will include your full peer review and any attached files.

**Do you want your identity to be public for this peer review?**  If you choose “no”, your identity will remain anonymous but your review may still be made public.

For information about this choice, including consent withdrawal, please see our Privacy Policy.

Reviewer #1: No

Reviewer #2: No

**********

[NOTE: If reviewer comments were submitted as an attachment file, they will be attached to this email and accessible via the submission site. Please log into your account, locate the manuscript record, and check for the action link "View Attachments". If this link does not appear, there are no attachment files.]

-->Figure Resubmissions:

While revising your submission, we strongly recommend that you use PLOS’s NAAS tool (https://ngplosjournals.pagemajik.ai/artanalysis) to test your figure files. NAAS can convert your figure files to the TIFF file type and meet basic requirements (such as print size, resolution), or provide you with a report on issues that do not meet our requirements and that NAAS cannot fix.

After uploading your figures to PLOS’s NAAS tool - https://ngplosjournals.pagemajik.ai/artanalysis, NAAS will process the files provided and display the results in the "Uploaded Files" section of the page as the processing is complete. If the uploaded figures meet our requirements (or NAAS is able to fix the files to meet our requirements), the figure will be marked as "fixed" above. If NAAS is unable to fix the files, a red "failed" label will appear above. When NAAS has confirmed that the figure files meet our requirements, please download the file via the download option, and include these NAAS processed figure files when submitting your revised manuscript.

# Author comment: Author response to Decision Letter 2

**DOI:** 10.1371/journal.pgph.0006720.r005

14 Apr 2026

Attachment Submitted filename: R2 TBMM PLOS GPH Response to reviewers.docx

# Peer review: Decision Letter 2

**DOI:** 10.1371/journal.pgph.0006720.r006

**Authors:** Graeme Hoddinott, Graeme Hoddinott, Graeme Hoddinott

5 May 2026

PGPH-D-25-01800R2

Tuberculosis comorbidities, multimorbidity and mortality amongst adults in South Africa: a prospective cohort study nested in a trial

PLOS Global Public Health

Dear Dr. Wingfield,

Thank you for submitting your manuscript to PLOS Global Public Health. After careful consideration, we feel that it has merit but does not fully meet PLOS Global Public Health’s publication criteria as it currently stands. Therefore, we invite you to submit a revised version of the manuscript that addresses the points raised during the review process.

EDITOR:

One of the reviewers has raised the point about the definitions of co- and multi-morbidity. Their reading of the "<!--StartFragment-->WHO framework on tuberculosis and comorbidities<!--EndFragment-->" is that "Multimorbidity is the presence of two or more concurrent disease or health conditions in a person with TB." They argue that this means having TB plus at least two other diseases / health conditions. Further, that the WHO definition for "Comorbidity" is having another (one other) concurrent disease or health condition alongside TB. The reviewer notes that you define "multimorbidity as two or more concurrent disease or health conditions in a single person", and "TB multimorbidity as two or more concurrent diseases or health conditions one of which is TB". There is therefore some divergence. What you are calling TB multimorbidity the WHO may be defining as TB comorbidity. And what you are defining as multimorbidity (two diseases / health conditions) concurrently is outside of the WHO definition for multimorbidity (three diseases; TB plus at least two others). The reviewer further notes that prior to the WHO definition, there has been a host of different definitions used in the literature which has caused much confusion and inability to compare / synthesize across the evidence base. Is it at all possible for you to engage this complexity to ensure that your (very important) manuscript does not leave readers similarly confused? Even if this is only in detailed noting of limitations and not a re-doing of any analysis. It may be helpful to reclassify some of what you are labelling as TB multimorbidity as TB co-morbidity as here this is overlap of definitions.

Multimorbidity: The presence of two or more concurrent diseases or health conditions in a single person.

-->TB Multimorbidity: The presence of two or more concurrent diseases or health conditions in a single person, one of which is TB.

Please submit your revised manuscript by Jun 04 2026 11:59PM. If you will need more time than this to complete your revisions, please reply to this message or contact the journal office at globalpubhealth@plos.org. When you're ready to submit your revision, log on to https://www.editorialmanager.com/pgph/ and select the 'Submissions Needing Revision' folder to locate your manuscript file.

Please include the following items when submitting your revised manuscript:

- A letter that responds to each point raised by the editor and reviewer(s). You should upload this letter as a separate file labeled 'Response to Reviewers'.
- A marked-up copy of your manuscript that highlights changes made to the original version. You should upload this as a separate file labeled 'Revised Manuscript with Track Changes'.
- An unmarked version of your revised paper without tracked changes. You should upload this as a separate file labeled 'Manuscript'.

Guidelines for resubmitting your figure files are available below the reviewer comments at the end of this letter.

As the corresponding author, your ORCID iD is verified in the submission system and will appear in the published article. PLOS supports the use of ORCID, and we encourage all coauthors to register for an ORCID iD and use it as well. Please encourage your coauthors to verify their ORCID iD within the submission system before final acceptance, as unverified ORCID iDs will not appear in the published article. *Only*  the individual author can complete the verification step; PLOS staff *cannot*  verify ORCID iDs on behalf of authors.

We look forward to receiving your revised manuscript.

Kind regards,

Graeme Hoddinott, Ph.D

Academic Editor

PLOS Global Public Health

Journal Requirements:

If the reviewer comments include a recommendation to cite specific previously published works, please review and evaluate these publications to determine whether they are relevant and should be cited. There is no requirement to cite these works unless the editor has indicated otherwise.

Please review your reference list to ensure that it is complete and correct. If you have cited papers that have been retracted, please include the rationale for doing so in the manuscript text, or remove these references and replace them with relevant current references. Any changes to the reference list should be mentioned in the rebuttal letter that accompanies your revised manuscript. If you need to cite a retracted article, indicate the article’s retracted status in the References list and also include a citation and full reference for the retraction notice.

Additional Editor Comments (if provided):

[Note: HTML markup is below. Please do not edit.]

Reviewers' comments:

Reviewer's Responses to Questions

**Comments to the Author**

1. If the authors have adequately addressed your comments raised in a previous round of review and you feel that this manuscript is now acceptable for publication, you may indicate that here to bypass the “Comments to the Author” section, enter your conflict of interest statement in the “Confidential to Editor” section, and submit your "Accept" recommendation.

Reviewer #1: All comments have been addressed

**********

2. Does this manuscript meet PLOS Global Public Health’s publication criteria? Is the manuscript technically sound, and do the data support the conclusions? The manuscript must describe methodologically and ethically rigorous research with conclusions that are appropriately drawn based on the data presented.

Reviewer #1: Yes

**********

3. Has the statistical analysis been performed appropriately and rigorously?

Reviewer #1: I don't know

**********

4. Have the authors made all data underlying the findings in their manuscript fully available (please refer to the Data Availability Statement at the start of the manuscript PDF file)?

The PLOS Data policy requires authors to make all data underlying the findings described in their manuscript fully available without restriction, with rare exception. The data should be provided as part of the manuscript or its supporting information, or deposited to a public repository. For example, in addition to summary statistics, the data points behind means, medians and variance measures should be available. If there are restrictions on publicly sharing data—e.g. participant privacy or use of data from a third party—those must be specified.

Reviewer #1: Yes

**********

5. Is the manuscript presented in an intelligible fashion and written in standard English?

PLOS Global Public Health does not copyedit accepted manuscripts, so the language in submitted articles must be clear, correct, and unambiguous. Any typographical or grammatical errors should be corrected at revision, so please note any specific errors here.

Reviewer #1: Yes

**********

6. Review Comments to the Author

Please use the space provided to explain your answers to the questions above. You may also include additional comments for the author, including concerns about dual publication, research ethics, or publication ethics. (Please upload your review as an attachment if it exceeds 20,000 characters)

Reviewer #1: Thank you for the revisions and improvments

**********

7. PLOS authors have the option to publish the peer review history of their article (what does this mean?). If published, this will include your full peer review and any attached files.

**Do you want your identity to be public for this peer review?**  If you choose “no”, your identity will remain anonymous but your review may still be made public.

For information about this choice, including consent withdrawal, please see our Privacy Policy.

Reviewer #1: No

**********

[NOTE: If reviewer comments were submitted as an attachment file, they will be attached to this email and accessible via the submission site. Please log into your account, locate the manuscript record, and check for the action link "View Attachments". If this link does not appear, there are no attachment files.]

Figure Resubmissions:

While revising your submission, we strongly recommend that you use PLOS’s NAAS tool (https://ngplosjournals.pagemajik.ai/artanalysis) to test your figure files. NAAS can convert your figure files to the TIFF file type and meet basic requirements (such as print size, resolution), or provide you with a report on issues that do not meet our requirements and that NAAS cannot fix.

After uploading your figures to PLOS’s NAAS tool - https://ngplosjournals.pagemajik.ai/artanalysis, NAAS will process the files provided and display the results in the "Uploaded Files" section of the page as the processing is complete. If the uploaded figures meet our requirements (or NAAS is able to fix the files to meet our requirements), the figure will be marked as "fixed" above. If NAAS is unable to fix the files, a red "failed" label will appear above. When NAAS has confirmed that the figure files meet our requirements, please download the file via the download option, and include these NAAS processed figure files when submitting your revised manuscript.

# Author comment: Author response to Decision Letter 3

**DOI:** 10.1371/journal.pgph.0006720.r007

3 Jun 2026

Attachment Submitted filename: R3 TBMM PLOSGPH Response to reviewers.docx

# Editor report: Decision Letter 3

**DOI:** 10.1371/journal.pgph.0006720.r008

**Authors:** Graeme Hoddinott, Graeme Hoddinott, Graeme Hoddinott, Graeme Hoddinott

8 Jun 2026

Tuberculosis comorbidities, multimorbidity and mortality amongst adults in South Africa: a prospective cohort study nested in a trial

PGPH-D-25-01800R3

Dear Dr. Wingfield,

We are pleased to inform you that your manuscript 'Tuberculosis comorbidities, multimorbidity and mortality amongst adults in South Africa: a prospective cohort study nested in a trial' has been provisionally accepted for publication in PLOS Global Public Health.

Before your manuscript can be formally accepted you will need to complete some formatting changes, which you will receive in a follow up email. A member of our team will be in touch with a set of requests.

Please note that your manuscript will not be scheduled for publication until you have made the required changes, so a swift response is appreciated.

IMPORTANT: The editorial review process is now complete. PLOS will only permit corrections to spelling, formatting or significant scientific errors from this point onwards. Requests for major changes, or any which affect the scientific understanding of your work, will cause delays to the publication date of your manuscript.

If your institution or institutions have a press office, please notify them about your upcoming paper to help maximize its impact. If they'll be preparing press materials, please inform our press team as soon as possible -- no later than 48 hours after receiving the formal acceptance. Your manuscript will remain under strict press embargo until 2 pm Eastern Time on the date of publication. For more information, please contact globalpubhealth@plos.org.

Thank you again for supporting Open Access publishing; we are looking forward to publishing your work in PLOS Global Public Health.

Best regards,

Graeme Hoddinott, Ph.D

Academic Editor

PLOS Global Public Health

***********************************************************

Reviewer Comments (if any, and for reference):
